# Na_v_1.7 is required for normal C-low threshold mechanoreceptor function in humans and mice

**DOI:** 10.1093/brain/awab482

**Published:** 2021-12-27

**Authors:** Steven J Middleton, Irene Perini, Andreas C Themistocleous, Greg A Weir, Kirsty McCann, Allison M Barry, Andrew Marshall, Michael Lee, Leah M Mayo, Manon Bohic, Georgios Baskozos, India Morrison, Line S Löken, Sarah McIntyre, Saad S Nagi, Roland Staud, Isac Sehlstedt, Richard D Johnson, Johan Wessberg, John N Wood, Christopher G Woods, Aziz Moqrich, Håkan Olausson, David L Bennett

**Affiliations:** Nuffield Department of Clinical Neurosciences, University of Oxford, Oxford OX3 9DU, UK; Center for Social and Affective Neuroscience, Department of Biomedical and Clinical Sciences, Linköping University, Linköping, Sweden; Center for Medical Image Science and Visualization, Linköping, Sweden; Nuffield Department of Clinical Neurosciences, University of Oxford, Oxford OX3 9DU, UK; Brain Function Research Group, School of Physiology, Faculty of Health Sciences, University of the Witwatersrand, Johannesburg, South Africa; Nuffield Department of Clinical Neurosciences, University of Oxford, Oxford OX3 9DU, UK; Institute of Neuroscience and Psychology, College of Medical, Veterinary and Life Sciences, University of Glasgow, Glasgow G12 8QQ, UK; Nuffield Department of Clinical Neurosciences, University of Oxford, Oxford OX3 9DU, UK; Nuffield Department of Clinical Neurosciences, University of Oxford, Oxford OX3 9DU, UK; Institute of Aging and Chronic Disease, University of Liverpool, L3 5DA Liverpool, UK; University Division of Anaesthesia, University of Cambridge, Cambridge NHS Foundation Trust Hospitals, Hills Road, Cambridge CB2 0QQ, UK; Center for Social and Affective Neuroscience, Department of Biomedical and Clinical Sciences, Linköping University, Linköping, Sweden; Aix-Marseille-Université, CNRS, Institute de Biologie du Développement de Marseille, UMR 7288, case 907, 13288 Marseille Cedex 09, France; Department of Cell Biology and Neuroscience, Rutgers, The State University of New Jersey, Piscataway, NJ 08854, USA; Nuffield Department of Clinical Neurosciences, University of Oxford, Oxford OX3 9DU, UK; Center for Social and Affective Neuroscience, Department of Biomedical and Clinical Sciences, Linköping University, Linköping, Sweden; Department of Physiology, University of Gothenburg, Gothenburg, Sweden; Center for Social and Affective Neuroscience, Department of Biomedical and Clinical Sciences, Linköping University, Linköping, Sweden; Center for Social and Affective Neuroscience, Department of Biomedical and Clinical Sciences, Linköping University, Linköping, Sweden; Department of Physiological Sciences, University of Florida College of Veterinary Medicine, Gainesville, FL, USA; Department of Psychology, University of Gothenburg, Gothenburg, Sweden; Department of Physiology, University of Gothenburg, Gothenburg, Sweden; Department of Physiological Sciences, University of Florida College of Veterinary Medicine, Gainesville, FL, USA; Department of Physiology, University of Gothenburg, Gothenburg, Sweden; Molecular Nociception Group, Wolfson Institute for Biomedical Research, University College London, Gower Street, London WC1E 6BT, UK; Cambridge Institute for Medical Research, School of Clinical Medicine, University of Cambridge, Cambridge, UK; Aix-Marseille-Université, CNRS, Institute de Biologie du Développement de Marseille, UMR 7288, case 907, 13288 Marseille Cedex 09, France; Center for Social and Affective Neuroscience, Department of Biomedical and Clinical Sciences, Linköping University, Linköping, Sweden; Nuffield Department of Clinical Neurosciences, University of Oxford, Oxford OX3 9DU, UK

**Keywords:** affective touch, C-low threshold mechanoreceptors, congenital insensitivity to pain, Na_v_1.7

## Abstract

Patients with bi-allelic loss of function mutations in the voltage-gated sodium channel Na_v_1.7 present with congenital insensitivity to pain (CIP), whilst low threshold mechanosensation is reportedly normal. Using psychophysics (*n* = 6 CIP participants and *n* = 86 healthy controls) and facial electromyography (*n* = 3 CIP participants and *n* = 8 healthy controls), we found that these patients also have abnormalities in the encoding of affective touch, which is mediated by the specialized afferents C-low threshold mechanoreceptors (C-LTMRs).

In the mouse, we found that C-LTMRs express high levels of Na_v_1.7. Genetic loss or selective pharmacological inhibition of Na_v_1.7 in C-LTMRs resulted in a significant reduction in the total sodium current density, an increased mechanical threshold and reduced sensitivity to non-noxious cooling. The behavioural consequence of loss of Na_v_1.7 in C-LTMRs in mice was an elevation in the von Frey mechanical threshold and less sensitivity to cooling on a thermal gradient.

Na_v_1.7 is therefore not only essential for normal pain perception but also for normal C-LTMR function, cool sensitivity and affective touch.

## Teaser

Middleton, Perini *et al*. show that the role of Na_v_1.7 extends beyond pain perception. Using a multidisciplinary, cross-species approach, they show that Na_v_1.7 is also essential for C-low threshold mechanoreceptor function in mice and humans, regulating pleasant touch, punctate discrimination and sensitivity to cooling.

## Introduction

Touch sensation is a critical component of the sensory system giving us the ability to detect, discriminate and explore our environment and also provides a substrate for social interaction. Low threshold mechanoreceptors (LTMRs) are a heterogenous group of sensory neurons which encode mechanical stimuli and can be classified according to their conduction velocity, stimulus-response function and the end-organs they innervate. Although the majority of C-fibre sensory afferents are nocioceptors and thermoceptors, an unmyelinated C-fibre population, termed C-low threshold mechanoreceptors (C-LTMRs, often termed CT-afferents in humans) were first discovered 80 years ago in cat^1^ and much later in humans.^2,3^ In humans, C-LTMRs respond to low threshold punctate indentations and have conduction velocities in the C-fibre range.^3^ Human C-LTMRs also underpin pleasant touch, a category of tactile perception, which until recently has been largely understudied. Yet, evidence now suggests that they are highly important for social contact, communication, relationships, pain relief and empathy for touch observed in others.^4–6^ These afferents respond to brushing stimuli between 1–10 cm/s and show an inverted U-shape relationship, with peak firing rates and peak perceived pleasantness seen at 3 cm/s.^7^ A sub-set of these afferents have also been shown to respond to cooling stimuli in humans.^2^

C-LTMRs have been identified in other mammals, including rodents.^8,9^ Several studies have identified molecular markers of rodent C-LTMRs (vGLUT3,^10^ Tafa4^11^ and IB4-GINIP+^12^), one of which is tyrosine hydroxylase (TH), a marker of all C-LTMRs.^13^ In addition, recent single-cell RNA sequencing studies have provided further evidence confirming that C-LTMRs have a very distinct transcriptional profile compared with other sensory neuron populations.^14,15^ The TH-positive population makes up ∼10% of all dorsal root ganglion (DRG) neurons, and sparse genetic labelling of TH-positive C-LTMRs revealed sensory endings innervating mouse hairy skin as longitudinal lanceolate endings surrounding hair follicles.^13^ C-LTMR sensory neurons also project centrally to the spinal cord and terminate in lamina IIi of the dorsal horn, where they synapse with distinct inhibitory (parvalbumin) and excitatory (PKCγ) interneuron populations.^13,16^ Li *et al*.^13^ also confirmed that TH-positive DRG neurons function like human C-LTMRs and have low mechanical thresholds, C-fibre range conduction velocities and respond to cooling stimuli. Because molecular identifiers of C-LTMRs have emerged relatively recently, there has been a dearth of studies into the role of sodium channel genes and their mutations in C-LTMRs in either humans or rodents. This is particularly relevant, as voltage-gated sodium channels (VGSCs) have emerged as important analgesic drug targets. Two recent studies suggest that C-LTMRs show significant expression of *SCN9A*,^15,17^ the gene encoding VGSC Na_v_1.7, which human genetics has strongly linked to nocioception and pain.^18^ Gain-of-function (GOF) mutations in Na_v_1.7 can result in painful conditions such as erythromelalgia, paroxysmal extreme pain disorder (PEPD), small fibre neuropathy and painful diabetic neuropathy,^19–23^ whilst bi-allelic loss-of-function (LOF) mutations lead to congenital insensitivity to pain (CIP), in which patients do not perceive pain in response to noxious mechanical, thermal or chemical stimuli.^24–26^ These striking psychophysical features are accompanied by a loss of functional C-nocioceptors (assessed using microneurography), highlighting Na_v_1.7 as an important modulator of the nocioceptive system.^27^ This sensory loss has been thought to be relatively selective, with an absence of pain perception, chemogenic itch and smell; touch and proprioceptive function were reportedly normal.^24–27^ This human genetic data, the relatively selective expression of Na_v_1.7 in the peripheral versus central nervous system and preclinical studies have made Na_v_1.7 an attractive druggable target to treat painful conditions.^28–32^ A number of small molecule blockers targeting Na_v_1.7 are currently in clinical development.

Whilst understandably there has been a focus linking Na_v_1.7 to human nocioception, C-LTMR function has not specifically been investigated to date. We have used a multi-disciplinary approach to answer this question by studying humans with LOF mutations in Na_v_1.7 alongside mice in which Na_v_1.7 has been ablated in C-LTMRs. We find that Na_v_1.7 LOF in humans not only leads to CIP, but also an impairment in affective touch, and that the stimulus-response function of C-LTMRs to mechanical and cooling stimuli is critically dependent on functional Na_v_1.7. Finally, we challenge the current dogma that therapeutics targeting Na_v_1.7 will act only on the nocioceptive system and that treatments may have undesired impacts on affective touch sensation.

## Materials and methods

### Humans

Six participants with bi-allelic LOF mutations in *SCN9A* and CIP [two males and four females, mean age = 35 years (SD = 11.02 years); Supplementary Table 1] took part in psychophysical testing and were compared with a large normative sample of healthy subjects,^4,33,34^ which included age- and sex-matched controls (*n* = 86, 45 females, age range = 16–60 years, mean age = 36 years, SD = 12.2 years). All of the participants exhibited the typical features of congenital insensitivity to pain with a history of never having experienced pain and multiple painless injuries such as burns and fractures. Facial EMG was collected in three participants (Participants 2, 3 and 4) and eight age-matched controls. Ethical approval was obtained by the ethics board of Linköping University (dnr 2014/341-31, dnr 2017/392-32, 2018/623-32 and 2017/485-31) and the National Research Ethics of the United Kingdom (Painful Channelopathies Study, NRES-UK reference: 12/LO/0017). Participants gave informed consent in accordance with the Declaration of Helsinki. See Supplementary material for detailed information about the human participants.

#### Affective touch testing: psychophysics

Single brush strokes were manually delivered to a 9 cm section of the forearm of each participant (*n* = 6 CIP participants and *n* = 86 healthy controls) using a soft 7 cm-wide brush. Thirty brush strokes were delivered in a distal to proximal direction at five different velocities in a pseudo-randomized order: 0.3 cm/s, 1 cm/s, 3 cm/s, 10 cm/s and 30 cm/s. Participants rated unpleasantness or pleasantness using a visual-analogue scale with the anchor points ‘unpleasant’ (−10) and ‘pleasant’ (+10). See Supplementary material for detailed information about the psychophysical tests.

Average scores per velocity per participant were entered in a 2 × 5 factorial ANOVA with ‘speed’ and ‘group’ as factors. In addition, for the healthy control participants, we assessed potential sex differences in a 2 × 5 factorial ANOVA with ‘speed’ and ‘sex’ as factors. *Post hoc* analysis was performed using Mann–Whitney tests. All data were analysed using the Statistical Package for the Social Sciences (SPSS Inc., Chicago, IL, USA).

#### Affective touch testing: facial electromyography

Participants (CIP participants 2, 3 and 4, *n* = 8 age- and sex-matched healthy controls) were fitted with surface electrodes placed above the eyebrow to measure corrugator supercilii (‘corrugator’) muscle region activity, and over the cheek, measuring zygomaticus major (‘zygomatic’) muscle region reactivity according to Fridlund and Cacioppo.^35^ Affective responses were assessed by measuring corrugator and zygomatic reactivity in response to each stimulus, quantified as mean EMG activation during the 6 s stimulus presentation minus the mean EMG activation during the 1 s before the stimulus was presented. Touch was administered using a soft 5 cm-wide brush applied to a 9 cm section of the forearm as detailed previously.^36,37^ The task consisted of four blocks; each block consisted of eight trials, four at each velocity, 3 cm/s (slow) and 30 cm/s (fast), with the velocity order within each block pseudo-randomized but not repeated more than three times. During the inter-trial intervals, participants were rated on a visual-analogue scale ‘How PLEASANT was the touch?’ or ‘How INTENSE was the touch?’ and could choose from −10 (extremely unpleasant) to +10 (extremely pleasant) or −10 (not at all intense) to +10 (extremely intense), respectively, using the mouse to move the visual-analogue scale slider. Within-group repeated measures ANOVAs with ‘speed’ as factor were performed on behavioural ratings and on facial muscle activity scores. Individual CIP participant’s scores were compared using independent-samples *t*-tests or Mann–Whitney tests when data were not normally distributed. See Supplementary material for detailed information on facial EMG testing.

### Animals

All mice were group-housed in individually ventilated cages, with free access to food and water, in humidity and temperature controlled rooms, with a 12 h light-dark cycle in a pathogen-free facility. All animal procedures adhered to the UK Home Office (Scientific Procedures) Act (1986) and were performed under a UK Home Office Project License. All animal experiments were carried out in accordance with University of Oxford Policy on the Use of Animals in Scientific Research. The work within this study also conforms to the ARRIVE guidelines.^38^ See Supplementary material for detailed information on mouse strains used.

#### Immunohistochemistry and *in situ* hybridization

Animals were deeply anaesthetized with pentobarbital and the blood cleared from all tissues by perfusing saline through the vascular system. Mice were then perfuse-fixed using 4% paraformaldehyde (PFA). Tissues were then collected and post-fixed in 4% PFA accordingly (DRG: 1–2 h, spinal cord: 24 h, skin: 1–2 h). All tissues were cryoprotected in 30% sucrose for a minimum of 48 h, followed by embedding the tissue and sectioning on a cryostat. (DRG: 12 μm, spinal cord: 20 μm, skin: 30 μm). Cultured cells were fixed with 4% PFA for 10 min and treated similarly to other tissues. Standard immunohistochemistry protocols were used.

*In situ* hybridization was performed using two methods; the first method (Fig. 3 and Supplementary Fig. 3C and D) was performed by following the user instructions for the RNAScope2.5 RED Chromogenic assay kit (Advanced Cell Diagnostics), with a Na_v_1.7mRNA specific probe (Cat no. 457641). The second method of *in situ* hybridization (Supplementary Fig. 3B) was performed using digoxigenin-labelled probes. Na_v_1.7 probes were hybridized overnight at 55°C and the slides incubated with the horseradish peroxidase anti-digoxigenin antibody (Roche). Final detection was achieved using a cy3 TSA plus kit (Perkin Elmer). For details of the immunohistochemistry, *in situ* hybridization and analyses, see Supplementary material.

#### Animal behaviour

Both male and female mice were used in this study, and mice were tested at a consistent time of day, in the same environment by the same experimenter. Mice were habituated to their testing environment and equipment prior to behavioural test days. The experimenter was blind to the animal genotype until after the behavioural analysis was complete.

#### Mechanical sensory testing

Mice were randomly assigned a test box (5 × 5 × 10 cm), which was elevated on a wire mesh base, and acclimatized to the equipment for 30–60 min. The plantar hind paws were tested using punctate von Frey hairs, brush and cotton swab stimuli. The dorsum of the hind paws was tested with sticky tape. Tactile acuity was assessed using the sandpaper tactile acuity test. See Supplementary material for details.

#### Thermal sensory testing

Mice were randomly selected from their home cages to randomize the order of thermal sensory assessment. Noxious thermal sensitivity was assessed using a 53°C hotplate, and thermal preference was assessed using a thermal gradient apparatus (6–54°C). For further details see Supplementary material.

#### Whole-cell patch clamp recordings

Voltage-clamp recordings using an Axopatch 200B amplifier and Digidata 1550 acquisition system (Molecular Devices) were performed at room temperature (21°C). Data were sampled at 20 kHz and low-pass filtered at 5 kHz. Series resistance was compensated 80–90% to reduce voltage errors. All data were analyzed by Clampfit 10 software (Molecular Devices). GFP/eYFP + DRG neurons (for cell culture methods see Supplementary material) were detected using an Olympus microscope with an inbuilt GFP filter set (470/40 × excitation filter, dichroic LP 495 mirror and 525/50 emission filter).

The protocol used (for solutions see Supplementary material) to assess peak voltage-gated Na^+^ currents consisted of a 20 ms test pulse to 0 mV, from a holding potential of −120 mV. The protocol used to assess the effect of PF-05089771 on Na^+^ currents consisted of a step from −120 mV to −75 mV for 8 s to inactivate a proportion of Na_v_1.7 channels, followed by a recovery step to −120 mV for 2 ms and a test pulse to 0 mV for 20 ms. This inactivation step was necessary, as PF-05089771 blocks Na_v_1.7 by binding in the inactivated state. We were guided by previous literature that −77 mV is the half inactivation of hNa_v_1.7.^39^ In both instances, three sweeps were taken, with an intersweep interval of 10 s, and the peak inward current during the test pulse was measured for each recording. Current/voltage (I/V) curves were generated from a series of incremental (Δ + 5 mV) 300 ms voltage steps from −80 to +35 mV, evoked every 10 s from a holding potential of −120 mV. Recordings were discarded if the series resistance was >15 MΩ or deviated by >20% during the recording. Linear leak subtraction was performed using P/4 leak subtraction.

#### *Ex vivo* skin-nerve preparation

The hind paw hairy skin and saphenous nerve was dissected and maintained in the inside-out orientation (hypodermis face up) in a recording chamber constantly perfused with synthetic interstitial fluid (SIF: 2.0 mM CaCl_2_, 5.5 mM glucose, 10 mM HEPES, 3.5 mM KCL, 0.7 mM MgSO_4_, 123 mM NaCl, 1.5 mM NaH_2_PO_4_, 9.5 mM Na-gluconate, 7.5 mM sucrose and 1 M NaOH; dH_2_O) at 32°C. The saphenous nerve was isolated using mineral oil (Sigma) in an adjacent chamber, de-sheathed and the nerve fibres teased apart and placed onto a silver recording electrode. Single-fibre receptive fields were located using a blunt probe and conduction velocity measured using pulsed supra-threshold electrical currents. C-LTMRs were identified on the basis of two main factors: conduction velocities below 1.2 m/s and v-Frey mechanical thresholds below 5.8 mN. All stimuli-evoked action potentials were visualized using an oscilloscope and recorded using a Powerlab 4.0 system in conjunction with LabChart v7.3 software (ADInstruments) (for stimuli see Supplementary material). The experimenter was blind to the animal genotype prior to the experiment until post analysis. For pharmacology, once C-LTMRs were identified in C57BL/6 mice, receptive fields were isolated using a metal ring, and the skin was stimulated pre and 10–12 min post application of 10 nM of the selective Na_v_1.7 channel blocker PF-05089771 or vehicle. The experimenter was blind to the treatment group until post analysis. On average, only one to two C-LTMRs were found per preparation; each preparation lasted for up to 8 h.

#### Computational modelling of C-LTMRs

The C-LTMR computational model used was previously described in detail by Zheng *et al*.^17^ and accessed from ModelDB (https://senselab.med.yale.edu/modeldb/, Accession No. 256632). CIP participant mutations from Participants 2, 3 and 4 were previously characterized by McDermott *et al*.^26^ in a heterologous expression system. The fold change decrease in the mutant Na_v_1.7 conductance, compared with the wild-type (WT) conductance, was calculated. In the C-LTMR model, the maximal Na_v_1.7 conductance was altered according to the conductance fold change due to each mutation (Supplementary Table 2) to create a new model for each mutation. The model was run in the naïve setting to model healthy controls without Na_v_1.7 mutations. The models applied successive current injections in increments of 1 pA in order to assess threshold excitability and 25 pA to assess suprathreshold excitability.

### Statistical analysis

All data were tested for normality using the D'Agostino–Pearson normality test and the appropriate parametric or non-parametric statistical tests were used accordingly. All statistical tests were two-tailed. Statistical comparisons were made using a Student’s *t*-test or Mann–Whitney U-test. In experimental groups in which multiple comparisons were made, one way or two-way ANOVA tests with appropriate *post hoc* tests were performed. All data are presented as mean ± the standard error of the mean (SEM) unless otherwise stated. Statistical significance is indicated as follows *P < 0.05, **P < 0.01 and ***P < 0.001. The statistical test used is reported in the appropriate figure legend. Graph Pad Prism 6 was used to perform statistical tests and graph data. Adobe illustrator CS5 was used to create schematics, and medical graphics were obtained from Smart Servier free medical art (smart.servier.com).

### Data availability

The data that support the findings of this study are available from the corresponding author upon reasonable request.

## Results

### CIP participants have an altered affective touch experience

**Figure 1:**
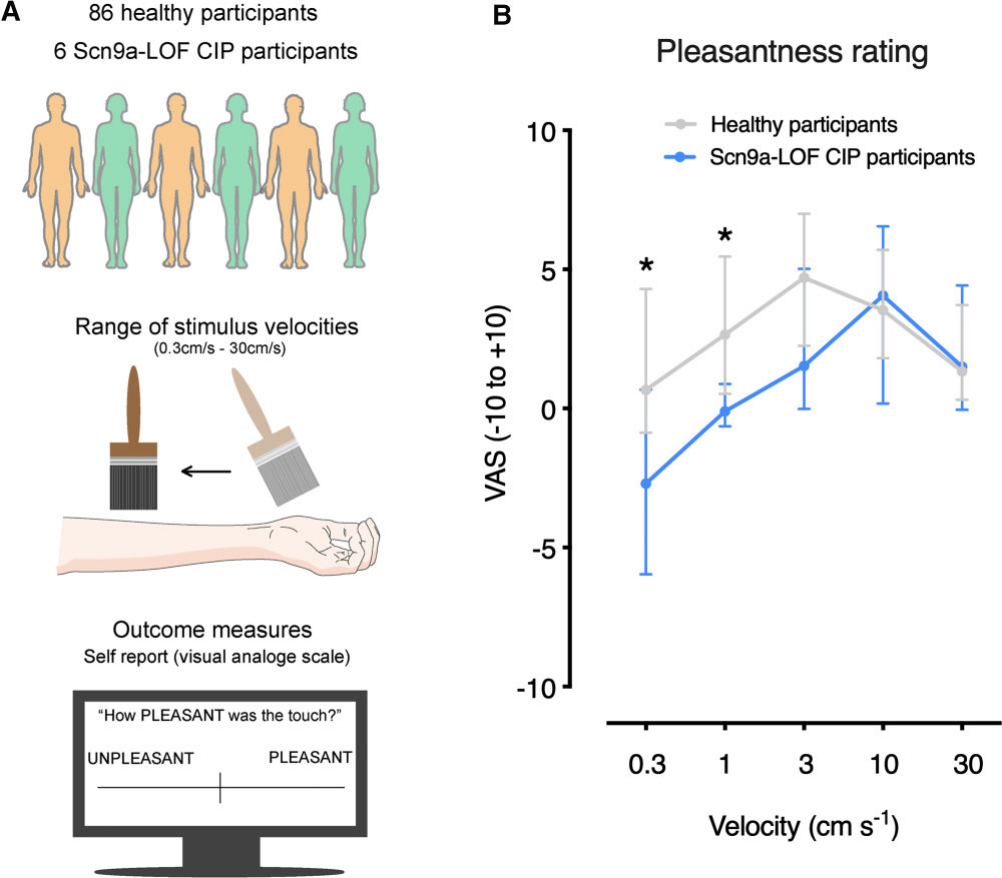
**SCN9A LOF participants perceive affective brush stimuli as less pleasant**. (**A**) A schematic outlining the two cohorts of participants that were recruited, the affective touch paradigm used and the self-report visual-analogue scale (VAS) outcome measure. (**B**) Touch pleasantness ratings across five stroking velocities in CIP participants with SCN9A mutations and healthy control participants. CIP participants found affective brush stimuli significantly less pleasant compared with healthy controls. A 2 × 5 repeated measures ANOVA revealed a significant main effect of speed [*F*(2.5,226.3) = 13.55, *P* < 0.001, $\eta_{p}^{2}$ = 0.13], a significant speed × group interaction [*F*(2.5, 226.3) = 3.80, *P* = 0.02, $\eta_{p}^{2}$ = 0.04] and a trend-level group effect [*F*(1,90) = 3.46, *P* = 0.07, $\eta_{p}^{2}$ = 0.04]. A *post hoc* Mann–Whitney test revealed a significant difference at 0.3 cm/s (U = 117, *P* = 0.03), 1 cm/s (U = 95, *P* = 0.01) and a trend for 3 cm/s (U = 149, *P* = 0.08). At 10 cm/s and 30 cm/s, the results were not significant (U = 252, *P* = 0.92 for 10 cm·s^−1^, U = 247, *P* = 0.86 for 10 cm/s). *P < 0.05. All data represented as median ± quartiles.

We investigated affective touch in six participants with LOF mutations in *SCN9A*, which principally results in CIP. Details about the participants recruited for this study are outlined in Supplementary Table 1. We presented healthy control participants and CIP participants with an affective touch paradigm (Fig. 1A), in which pleasantness was rated in response to brushing the forearm at different velocities. The self-reported scores from a large sample of healthy participants were consistent with previous literature demonstrating an inverted U-shaped visual-analogue scale pleasantness pattern with stimulus speed and the optimal brushing speed being 3 cm/s (Fig. 1B).^4,7,33,34,40^ No sex differences were identified in healthy participants (Supplementary Fig. 1). The CIP participant group reported significantly lower pleasantness ratings for slow brushing touch, and they did not show the classical U-shaped visual-analogue scale score response (Fig. 1B and Supplementary Fig. 1). The change in self-reported pleasantness in the CIP participant group was specific to slow brushing speeds and no difference was observed for faster speeds (10 cm/s and 30 cm/s) (Fig. 1B and Supplementary Fig. 1). This suggests that mutations in Na_v_1.7 have an impact on the affective perception of gentle touch.

**Figure 2:**
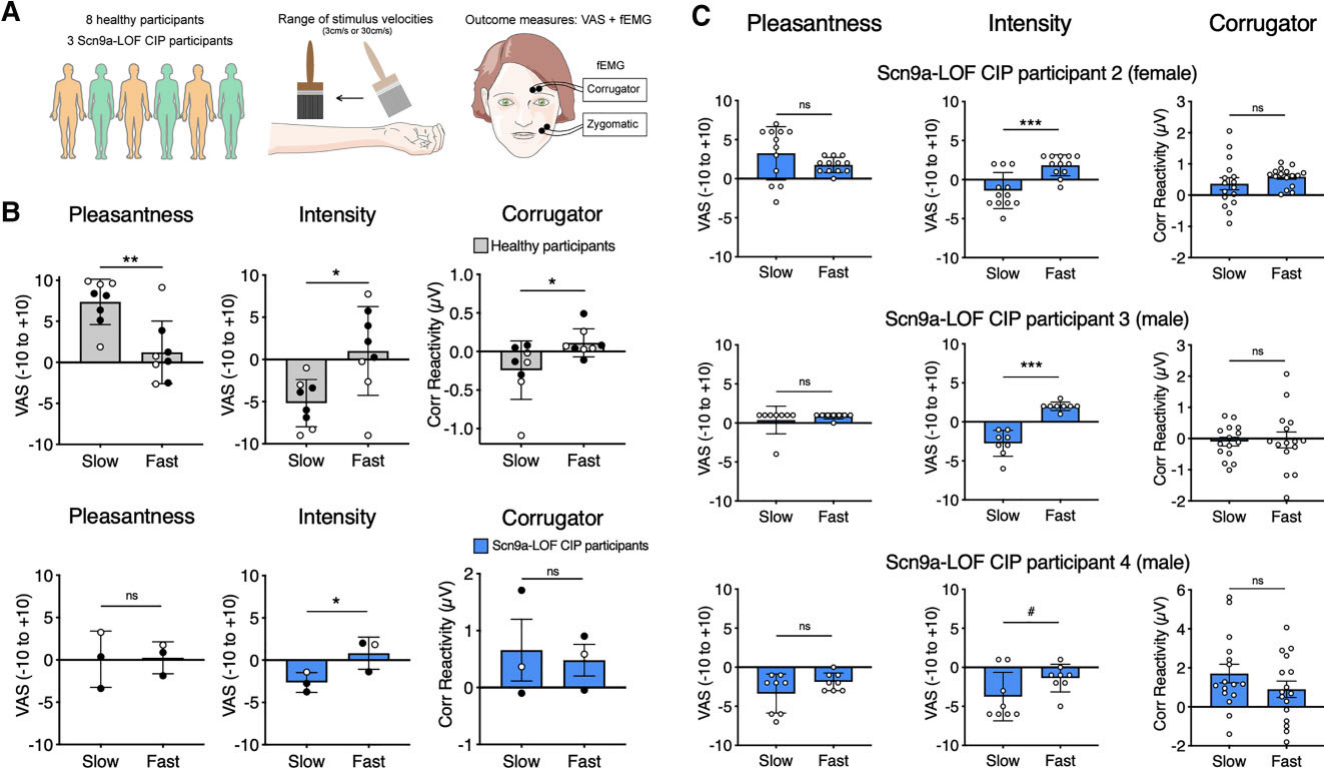
**SCN9A LOF participants show atypical functional EMG responses to affective touch stimuli**. (**A**) Study population, stimuli and measurements. (**B**) The average visual-analogue scale (VAS) ratings of pleasantness, intensity and average facial EMG (corrugator) responses to slow (3 cm/s) or fast (30 cm/s) brush stroking on the forearm from eight healthy control participants and three CIP participants. Black circles reflect mean values for male participants. Pleasantness: Healthy control participants rated slow bush as significantly more pleasant than fast brush [main effect of speed in healthy controls: *F*(1,7) = 23.8, *P* = 0.002, $\eta_{p}^{2}$ = 0.77]. CIP participants did not rate slow brush as more pleasant than fast brush [main effect of speed in CIP participants: *F*(1,2) = 0.04, *P* = 0.87, $\eta_{p}^{2}$ = 0.02]. Intensity: Healthy control and CIP participants rated fast brush as significantly more intense than slow brush [main effect of speed in healthy controls: *F*(1,7) = 9.21, *P* = 0.02, $\eta_{p}^{2}$ = 0.57; main effect of speed in CIP participants: *F*(1,2) = 24.87, *P* = 0.04, $\eta_{p}^{2}$ = 0.92]. Corrugator: In healthy controls there was significantly greater corrugator activity elicited from fast brush stimuli and a reduction in activity elicited by slow brush stimuli [*F*(1,7) = 8.59, *P* = 0.02, $\eta_{p}^{2}$ = 0.55]. This was not observed in CIP participants [*F*(1,2) = 0.31, *P* = 0.63, $\eta_{p}^{2}$ = 0.13]. Zygomatic: There was no significant effect of brushing velocity on zygomatic activity in either group [not illustrated; healthy controls: *F*(1,7) = 3.77, *P* = 0.093); CIP participants: *F*(1,2) = 3.25, *P* = 0.21]. (**C**) Trial-by-trial touch ratings and facial EMG responses for individual CIP participants. CIP participant 2, 3 and 4 all reported no difference in pleasantness between slow and fast brushing velocities [CIP 2: *t*(22) = 1.46, *P* = 0.16; CIP 3: *U* = 31, *P* = 0.9; CIP 4: *t*(14) = 1.55, *P* = 0.14]. CIP participants 2 (female) and 3 (male) rated fast touch as more intense than slow touch [CIP 2: *t*(22) = 4.21, *P* < 0.001; CIP 3: *t*(14) = 7.67, *P* < 0.001]. Similarly, CIP participant 4 reported slow touch as marginally less intense than fast [CIP 4: *t*(14) = 1.88, *P* = 0.08]. In CIP participants 2, 3 and 4, there was no velocity-based difference in corrugator activity [CIP 2: *t*(30) = 1.06; *P* = 0.30; CIP 3: *t*(28) = 0.19; *P* = 0.85; CIP 4: *t*(30) = 1.27, *P* = 0.22] or zygomatic activity (not illustrated) [CIP 2: *t*(27) = 0.015, *P* = 0.98; CIP 3 : *t*(27) = 0.76, *P* = 0.45; CIP 4: *t*(30) = 0.35; *P* = 0.73]. Asterisks reflect within-group and within-subject analyses, *P < 0.05, **P < 0.01, ***P < 0.001, ^#^*P* < 0.1. All data are represented as mean ± SD.

An extended examination, that included facial EMG as an indicator of emotional responses, was conducted on three of the CIP participants (Participants 2, 3 and 4) and in eight control participants (Fig. 2A). Again, consistent with Fig. 1B and previous studies,^4,7,33,34,40^ control participants rated slow stroking touch as significantly more pleasant and less intense than fast stroking touch (Fig. 2B). Facial EMG in healthy control participants revealed that pleasant slow stroking was associated with a relaxation of the corrugator (frowning) facial muscle, whereas the less pleasant fast stroking was associated with a contraction of the corrugator muscle (Fig. 2B).^36,37,41^ In contrast, the CIP participants showed no consistent corrugator activity to slow or fast brushing (Fig. 2B and C). Despite not rating either stimulus as more or less pleasant, the CIP participants were able to discriminate stimulus intensity (Fig. 2B and C).

Thus, taken together, the *SCN9A* mutations influenced not only the perception of touch pleasantness but also the emotional reactions to touch, as measured using facial EMG. As expected, the SCN9A mutations did not influence basic sensory-discriminative perception of brush stimuli.

#### C-LTMRs express Na_v_1.7

**Figure 3:**
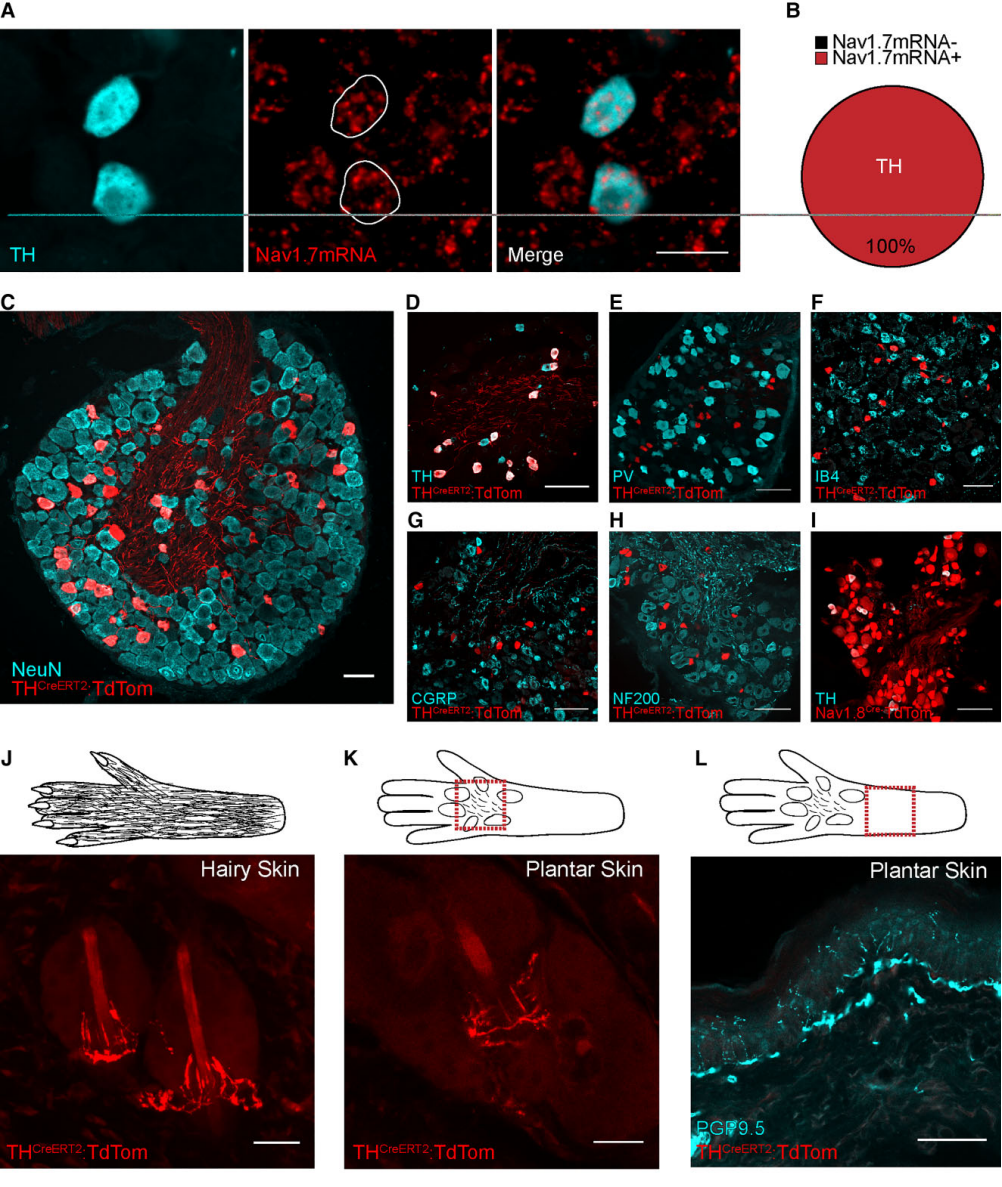
**Rodent C-LTMRs express Na_v_1.7, which innervate hind-paw hairy and plantar skin**. (**A**) *In situ* hybridization of L4 DRG sections showing Na_v_1.7 mRNA-positive cell co-localization with TH, a C-LTMR marker. Scale bars = 25 µm. (**B**) All (100%) TH-positive C-LTMRs expressed Na_v_1.7 mRNA (226/226 cells from three mice). (**C**) Example of genetically labelled C-LTMRs using the TH^CreERT2^ mouse line crossed with the Cre-dependent reporter (tdTomato). Scale bar = 50 µm. (**D**) Co-localization of genetically labelled C-LTMRs and TH antibody-labelled C-LTMR cell bodies in the DRG. TH^CreERT2^-positive C-LTMRs are a largely non-overlapping population and minimal co-localization was seen between parvalbumin (PV) (**E**), IB4 (**F**), CGRP (**G**) and NF200 (**H**). (**I**) Almost all TH-positive C-LTMRs co-express Na_v_1.8 as seen by co-localization of genetically labelled Na_v_1.8 afferents (Na_v_1.8^Cre^TdTom) and the TH antibody. **D**–**I** scale bar = 100 µm. (**J**) tdTomato-labelled C-LTMRs forming longitudinal lanceolate endings around hair follicles in hind-paw hairy skin. Scale bar = 25 µm. (**K**) tdTomato-positive C-LTMR innervating the hair follicles found on the plantar surface (located between the running pads) of mice. Scale bar = 25 µm. (**L**) TH-positive C-LTMRs do not terminate in the skin as PGP9.5+ epidermal free nerve endings. Scale bar = 50 µm.

Na_v_1.7 is highly expressed in the peripheral nervous system, with restricted expression within the CNS in sub-cortical structures, including the thalamus, medial amygdala, hypothalamus and the axons of the olfactory epithelium projecting to the olfactory bulb.^42,43^ Na_v_1.7 is highly expressed in C-nocioceptors.^44^ It has been shown through mRNA sequencing of the C-LTMR population that they also express Na_v_1.7.^45^ Using the dataset provided by Reynders *et al*.,^45^ we identified *SCN9A* expression in three sensory neuron populations: C-LTMRs (GINIP+/IB4−), non-peptidergic nocioceptors (GINIP+/IB4+) and all other DRG neurons (IB4−/GINIP−), with the highest reads per kilobase of transcript per million mapped reads (RPKM) seen in C-LTMRs (Supplementary Fig. 2). To validate these sequencing results, we carried out *in situ* hybridization combined with immunohistochemistry, and Na_v_1.7mRNA was indeed present in the C-LTMR population (GINIP+/IB4−) (Supplementary Fig. 2). We also found that 99.4% of peptidergic nocioceptors and 82.6% of myelinated fibres express Na_v_1.7 mRNA (Supplementary Fig. 2). More recently, Zheng *et al*.^17^ carried out deep sequencing of seven transgenically-labelled sensory neurons populations, including C-LTMRs. This work demonstrated that C-LTMRs identified by the expression of TH express Na_v_1.7 mRNA, and surprisingly this expression is highest in C-LTMRs as compared with seven other sensory neuron populations, including nocioceptors. To validate these sequencing results from Zheng *et al*.^17^ and ask what proportion of C-LTMRs express Na_v_1.7, we carried out *in situ* hybridization and immunohistochemistry and found that 100% of TH-positive C-LTMRs express Na_v_1.7 mRNA (Fig. 3A and B).

#### The TH^CreERT2^ transgenic mouse efficiently targets C-LTMRs that express Na_v_1.7

We characterized the TH^CreERT2^ line generated by Abraira *et al*.^46^ as a means to target the C-LTMR population. This line was used previously to demonstrate C-LTMR central projections terminating in lamina IIi of the dorsal horn. We bred the TH^CreERT2^ line with a Cre-dependent tdTomato reporter line and induced tdTomato expression in adulthood to characterize the C-LTMR population at the level of the DRG, spinal cord and skin. We showed that the labelled C-LTMR population makes up 4.7 ± 0.5% of lumbar and 9.8 ± 1.9% of thoracic DRG neurons (Fig. 3C), similar to previous studies.^13^ We confirmed that these neurons are indeed small, with an average neuronal area of 238.6 ± 8.3 µm^2^ (an area <490 µm^2^ denotes a small DRG neuron with a diameter of 25 µm). We showed that the TH^CreERT2^ line is ∼80% efficient at targeting the population when tamoxifen is given in adulthood (Fig. 3C and Supplementary Fig. 3). The transgenically-labelled TH-positive C-LTMRs form a distinct non-overlapping population that do not express/bind, parvalbumin (PV), IB4, CGRP or NF200, and all C-LTMRs express the VGSC Na_v_1.8 (Fig. 3D–I, Supplementary Fig. 3).^13,17^ We have also shown consistent and expected lamina IIi tdTomato C-LTMR terminations in the dorsal horn of the spinal cord (Supplementary Fig. 3) as reported previously.^46^ We assessed labelled sensory ending structures in the skin and identified longitudinal lanceolate endings associated with hair follicles on hind paw hairy skin (Fig. 3J). It has recently been reported that some species of rodent (including C57BL/6 strains) have hair follicles located between the running pads of their paws, which have been evolutionary conserved.^47^ Interestingly, we found that C-LTMR sensory endings are also present on the plantar surface of rodent glabrous skin and indeed innervate hairs located between rodent running pads (Fig. 3K). While further evidence is needed in humans, a recent microneurography study identified a small number of mechanosensitive units on glabrous skin with delayed responses to mechanical stimulation, suggesting that they may have C-LTMR characteristics.^48^ Finally, we did not see any labelled epidermal small fibres (nocioceptors) (Fig. 3L). Taken together, all C-LTMRs express Na_v_1.7 mRNA and the TH^CreERT2^ line first published by Abraira *et al.*^46^ is a good model for studying C-LTMR function.

#### Genetic loss of Na_v_1.7 in rodent and human C-LTMRs results in mechanical and cooling deficits

**Figure 4:**
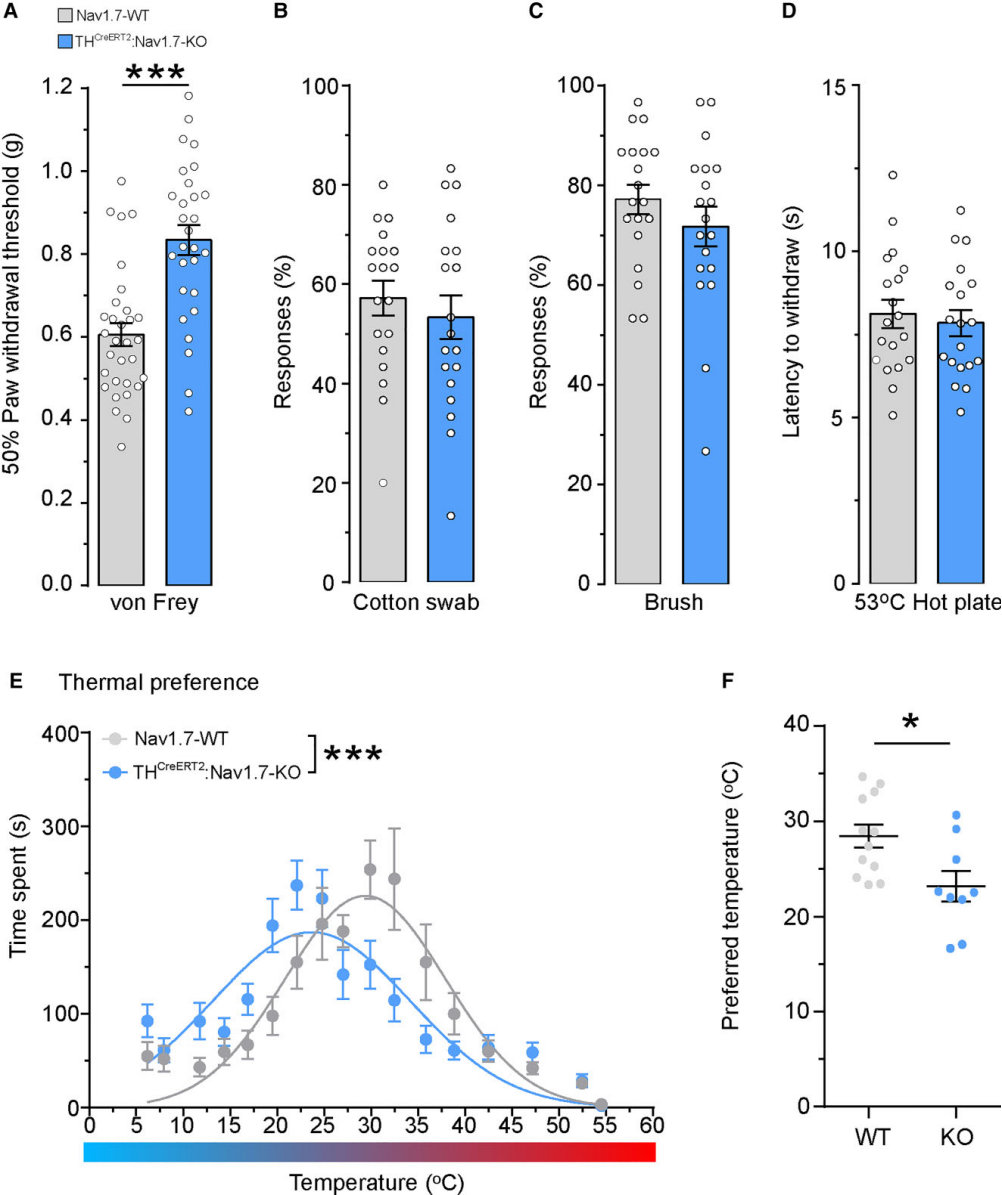
**The genetic loss of Na_v_1.7 in rodent C-LTMRs results in mechanical hyposensitivity and abnormalities in cooling detection**. Acute sensory testing of mice lacking Na_v_1.7 in the TH-positive C-LTMR population (blue: TH^CreERT2^:Na_v_1.7-KO) compared with controls (grey: Na_v_1.7-WT). (**A**) C-LTMR-specific deletion of Na_v_1.7 results in significant hyposensitivity to punctate mechanical von Frey stimuli [WT: *n* = 31 mice, KO: *n* = 28 mice, two-tailed Student’s unpaired *t*-test, *t*(57) = 5.062, *P* < 0.0001, ***]. (**B**) The number of responses to a cotton swab or (**C**) to a brush are not affected by the loss of Na_v_1.7 in rodent C-LTMRs [WT: *n* = 19 mice, KO: *n* = 19 mice, two-tailed Student’s unpaired *t*-test, *t*(36) = 0.690, *P* = 0.49 and *t*(36) = 1.09, *P* = 0.28, respectively, n.s]. (**D**) Mice specifically lacking Na_v_1.7 in C-LTMRs showed no changes in their latency to withdraw from a noxious 53°C hotplate. [WT: *n* = 19 mice, KO: *n* = 19 mice, two-tailed Student’s unpaired *t*-test, *t*(36) = 0.470, *P* = 0.64, n.s]. (**E**) Mice lacking Na_v_1.7 in C-LTMRs spent more time in cooler zones during 0–30 min of the thermal gradient test. The non-linear regression Gaussian fitted curves are significantly different between WT and KO mice, with KO mice showing a leftward shift toward colder temperatures [WT: *n* = 12 mice, KO: *n* = 9 mice, non-linear regression F-test, *F*(3,351) = 12.95, *P* = < 0.0001, ***]. (**F**) The preferred temperature (the average temperature at which most time was spent) was significantly lower in KO mice compared with WT mice during 0–30 min of a thermal gradient test [WT: *n* = 12 mice, KO: *n* = 9 mice, two-tailed Student’s unpaired *t*-test, *t*(19) = 2.689, *P* = 0.0145, *]. All data represented as mean ± SEM. *P < 0.05, ***P < 0.001.

To understand the role of Na_v_1.7 in mouse C-LTMRs, we generated a conditional C-LTMR-specific Na_v_1.7 knock-out (KO) mouse, using the previously discussed TH^CreERT2^ model crossed with a floxed Na_v_1.7 mouse line. Following administration of tamoxifen and the conditional KO of Na_v_1.7, we conducted an array of behaviour assays to profile sensory function. We found that mice lacking Na_v_1.7 in C-LTMRs exhibit a small but significant hyposensitivity to punctate mechanical stimuli (Fig. 4A). This finding led to re-examination of CIP participant 4 and their ability to discriminate between low-force punctate monofilaments. Consistently, the ability to discriminate between low force monofilaments was reduced in CIP participant 4 compared with 20 healthy control participants (Supplementary Fig. 4A). In addition, single-unit microneurography recordings in healthy participants demonstrated that human C-LTMRs can encode low-force punctate stimuli (Supplementary Fig. 4B).

In our conditional KO mouse, there was no difference in the number of responses to light brush stimuli as measured by a cotton swab, brush or sticky tape (Fig. 4B and C, Supplementary Fig. 5A). In addition, loss of Na_v_1.7 specifically in C-LTMRs did not affect tactile acuity tasks (Supplementary Fig. 5B–E). As expected, the loss of Na_v_1.7 in C-LTMRs did not affect the latency to withdraw from a nocioceptive hotplate (Fig. 4D). Mouse and human C-LTMRs are known to respond to cooling stimuli, and we have previously found that CIP participants show hyposensitivity to cold and cool stimuli (Supplementary Fig. 4C). We therefore sought to assess the loss of Na_v_1.7 in cold stimuli coding. We allowed mice to explore a temperature gradient apparatus freely, which ranged from 6–54°C for 30 min. Na_v_1.7-WT mice had a bell shaped (inverted U-shaped) response to the thermal gradient apparatus while TH^CreERT2^:Na_v_1.7-KO mice spent more time in cooler zones as seen by a significant leftward shift in the non-linear regression Gaussian curve (Fig. 4E), suggestive of a deficit in cool detection. Additionally, there was a ∼5°C reduction in the average preferred temperature (the temperature at which mice spent most of their time) in TH^CreERT2^:Na_v_1.7-KO mice (23.16 ± 1.59°C) compared with the WT mice (28.45 ± 1.21°C) (Fig. 4F). We also analysed our behavioural data in a sex-dependent manner, as previously it has been reported that mechanisms underlying thermal preference can differ between male and female mice.^49^ The mechanical and thermal phenotypes were present and consistent in both male and female mice (Supplementary Fig. 6A–G). Therefore, we concluded that the behavioural consequence of Na_v_1.7 loss of function in C-LTMRs is not sexually dimorphic. These data showing that the genetic loss of Na_v_1.7 in rodent and human C-LTMRs results in mechanical and cool sensory deficits illustrate that Na_v_1.7 is necessary for normal C-LTMR function.

#### C-LTMRs lacking Na_v_1.7 are hypo-excitable

**Figure 5:**
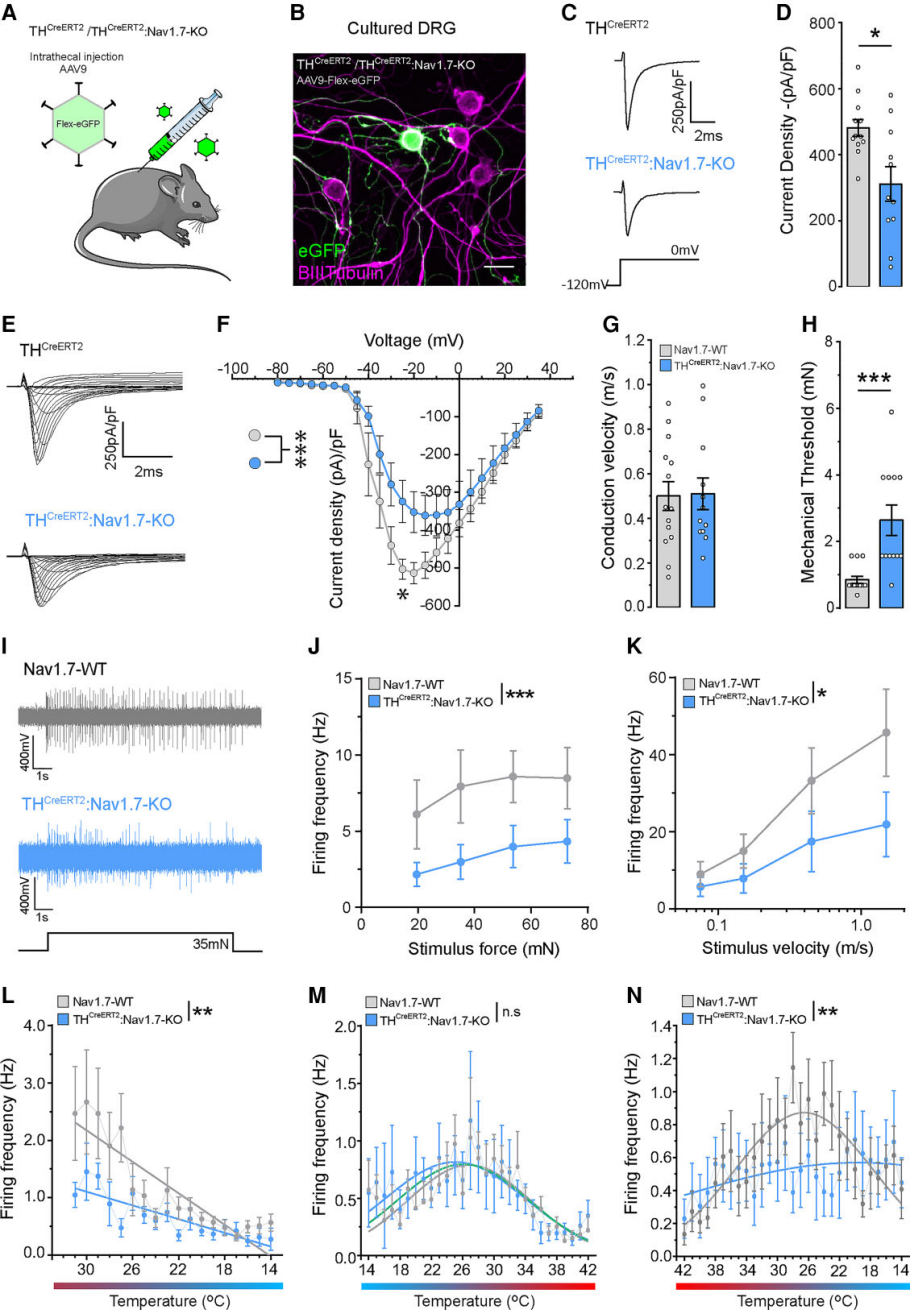
**Rodent C-LTMRs that lack Na_v_1.7 show smaller sodium currents and hypo-excitability**. (**A**) TH^CreERT2^ (control) or TH^CreERT2^Na_v_1.7^flox/flox^ (TH^CreERT2^Na_v_1.7-KO) mice received and intrathecal injection of AAV.Flex.eGFP to target C-LTMRs prior to tamoxifen administration. Subsequent tamoxifen injection initiated simultaneous eGFP expression and Na_v_1.7 ablation. (**B**) Virally targeted C-LTMRs were cultured, eGFP expression used to identify the population and voltage-clamp used to analyse sodium currents in both control and TH^CreERT2^Na_v_1.7-KO mice. (**C**) Example traces of recorded total sodium currents in eGFP-positive C-LTMRs from TH^CreERT2^ and TH^CreERT2^Na_v_1.7-KO mice. (**D**) C-LTMRs lacking Na_v_1.7 had a significantly reduced sodium current density compared with control C-LTMRs (TH^CreERT2^*n* = 12 cells, TH^CreERT2^Na_v_1.7-KO *n* = 11 cells. Mann–Whitney U-test, U = 28, *P* < 0.018, *). (**E**) Example sodium current traces from TH^CreERT2^ and TH^CreERT2^Na_v_1.7-KO C-LTMRs to determine the sodium current-voltage (I/V) relationship. (**F**) Quantification of the I/V relationship displayed as I/V curves. C-LTMRs from TH^CreERT2^Na_v_1.7-KO mice showed a significantly smaller I/V curve compared with C-LTMRs from TH^CreERT2^ mice (TH^CreERT2^: *n* = 12 cells, TH^CreERT2^Na_v_1.7-KO: *n* = 11 cells. Two-way ANOVA, *F*(1,432) = 24.05, *P* < 0.0001, ***, with Sidak–Holm *post hoc* test, −25 pA, *t*(432) = 3.342, *P* = 0.021, *). (**G**) Single fibre recordings from the mouse skin-nerve (saphenous) preparation comparing recordings from Na_v_1.7-WT (grey) and TH^CreERT2^:Na_v_1.7-KO (blue) mice. C-LTMR conduction velocities were normal and comparable between both WT and C-LTMRs lacking Na_v_1.7. [WT: *n* = 14 units, KO: *n* = 12 units, two-tailed Student’s unpaired *t*-test, *t*(24) = 0.103, *P* > 0.91, n.s.] (**H**) The mechanical thresholds of TH^CreERT2^:Na_v_1.7-KO C-LTMRs were significantly higher than Na_v_1.7-WT control C-LTMRs [WT: *n* = 14 units, KO: *n* = 12 units, two-tailed Student’s unpaired *t*-test, *t*(24) = 4.070, *P* = 0.0004,***] (**I**) Example trace of evoked action potentials in response to a supra-threshold mechanical stimulus applied to a single Na_v_1.7-WT and TH^CreERT2^:Na_v_1.7-KO C-LTMR receptive field. (**J**) The increasing force stimulus-response function showing that C-LTMRs lacking Na_v_1.7 were significantly hypo-excitable to supra-threshold stimuli compared with control C-LTMRs [WT: *n* = 14 units, KO: *n* = 12 units, two-way ANOVA, *F*(1,95) = 11.87, *P* = 0.0008, ***]. (**K**) The increasing velocity stimulus-response function of Na_v_1.7-WT and TH^CreERT2^:Na_v_1.7-KO C-LTMRs. C-LTMRs lacking Na_v_1.7 were hypo-excitable with a significantly reduced firing frequency to dynamic stimuli [WT: *n* = 14 units, KO: *n* = 12 units, two-way ANOVA, *F*(1,96) = 6.212, *P* = 0.014, *]. (**L**) The 31–14°C cooling stimulus-response of Na_v_1.7-WT and TH^CreERT2^:Na_v_1.7-KO C-LTMRs. C-LTMRs lacking Na_v_1.7 were hypo-excitable to cooling stimuli. The linear regression fitted slopes are significantly different between WT and KO mice [WT: *n* = 10 units, KO: *n* = 8 units, linear regression F-test, *F*(1,306) = 9.32, *P* = 0.0024, **]. (**M**) The 14–42°C warming stimulus-response of Na_v_1.7-WT and TH^CreERT2^:Na_v_1.7-KO C-LTMRs were similar. The non-linear regression Gaussian fitted curves are not significantly different between WT and KO mice, both groups and share a common curve (green) [WT: *n* = 10 units, KO: *n* = 8 units, non-linear regression F-test, *F*(3,504) = 0.763, *P* = 0.515, n.s.]. (**N**) The 42–14°C cooling stimulus-response of Na_v_1.7-WT and TH^CreERT2^:Na_v_1.7-KO C-LTMRs. C-LTMRs lacking Na_v_1.7 are hypo-excitable to cooling stimuli. The non-linear regression Gaussian fitted curves are significantly different between WT and KO mice [WT: *n* = 10 units, KO: *n* = 8 units, non-linear regression F-test, *F*(3,508) = 5.106, *P* = 0.0017, **]. All data represented as mean ± SEM. *P < 0.05, **P < 0.01, ***P < 0.001.

Given the changes in sensory behaviour in the TH^CreERT2^:Na_v_1.7-KO mice, we wanted to confirm our C-LTMR Na_v_1.7 KO and investigate the contribution of Na_v_1.7 to the total sodium currents in this population. We labelled the C-LTMR population using the Cre recombinase-dependent virus, AAV9.Flex.eGFP, giving us the ability to study C-LTMRs *in vitro*. We performed intrathecal injections of the reporter virus into TH^CreERT2^ and TH^CreERT2^:Na_v_1.7^flox/flox^ mice and administered tamoxifen 1 week later to initiate simultaneous eGFP expression and Na_v_1.7 ablation (Fig. 5A). Four weeks following tamoxifen dosing, we cultured lumbar DRG neurons from injected animals and performed voltage clamp analysis and single cell qPCR on eGFP-positive cells (Fig. 5B). Using single cell qPCR, we confirm that the Na_v_1.7 mRNA transcript level is significantly reduced in TH^CreERT2^:Na_v_1.7-KO C-LTMRs (Supplementary Fig. 7). Voltage clamp recordings showed a reduced peak inward current upon membrane depolarization to 0 mV (Fig. 5C and D) and lower current densities across a range of voltages at which voltage-gated sodium channels are known to activate (Fig. 5E and F) in C-LTMRs that lack Na_v_1.7 compared with WT neurons.

Due to Na_v_1.7’s large contribution to sodium currents in C-LTMRs, we next examined whether Na_v_1.7 directly regulated C-LTMR excitability. We performed single-fibre primary afferent characterization of C-LMTRs in hind paw hairy skin from Na_v_1.7-WT and TH^CreERT2^:Na_v_1.7-KO mice. The conduction velocity of recorded C-LTMRs were within the mouse C-fibre range (<1.2 m/s) and comparable between both WT and KO mice (Fig. 5G). However, there was a significant increase in the mechanical thresholds of TH^CreERT2^:Na_v_1.7-KO C-LTMRs compared with those recorded from Na_v_1.7-WT mice (Fig. 5H). We next analysed the stimulus response functions of C-LTMRs to suprathreshold punctate mechanical stimuli and saw that C-LTMRs lacking Na_v_1.7 fire less and display significant hypo-excitability (Fig. 5I and J). We also investigated the stimulus response functions in response to repeated punctate mechanical stimuli, where each stimulus (which is a downward indentation of the skin) increases its velocity. Rodent C-LTMRs from TH^CreERT2^:Na_v_1.7-KO mice exhibited a reduced firing frequency and are hypo-excitable to moving punctate stimuli compared with Na_v_1.7-WT mice (Fig. 5K).

Due to the mouse behavioural and human psychophysical data demonstrating cool detection abnormalities, we directly analysed the temperature sensibility of mouse C-LTMRs (Fig. 5L–N). Using three temperature ramps restricted to identified C-LTMR receptive fields, their response to cooling and warming stimuli was assessed in detail. The first temperature ramp started at 31°C (skin temperature) and cooled the receptive field to 14°C (Fig. 5L), the second ramp warmed the receptive field from 14°C to 42°C (Fig. 5M) and finally the third ramp cooled the receptive field from 42°C to 14°C (Fig. 5N). Our data demonstrated that WT C-LTMRs respond to both cooling and warming stimuli with a stimulus response that resembles an inverted U-shaped response, with a maximal firing at ∼27–28°C. However, C-LTMRs lacking functional Na_v_1.7 responded less and exhibited hypo-excitability to cooling stimuli (Fig. 5L–N). Collectively, we demonstrated that Na_v_1.7 is a key regulator of C-LTMR excitability in response to mechanical and cool stimuli.

#### Small molecule blockade of Na_v_1.7 reduces C-LTMR excitability

**Figure 6:**
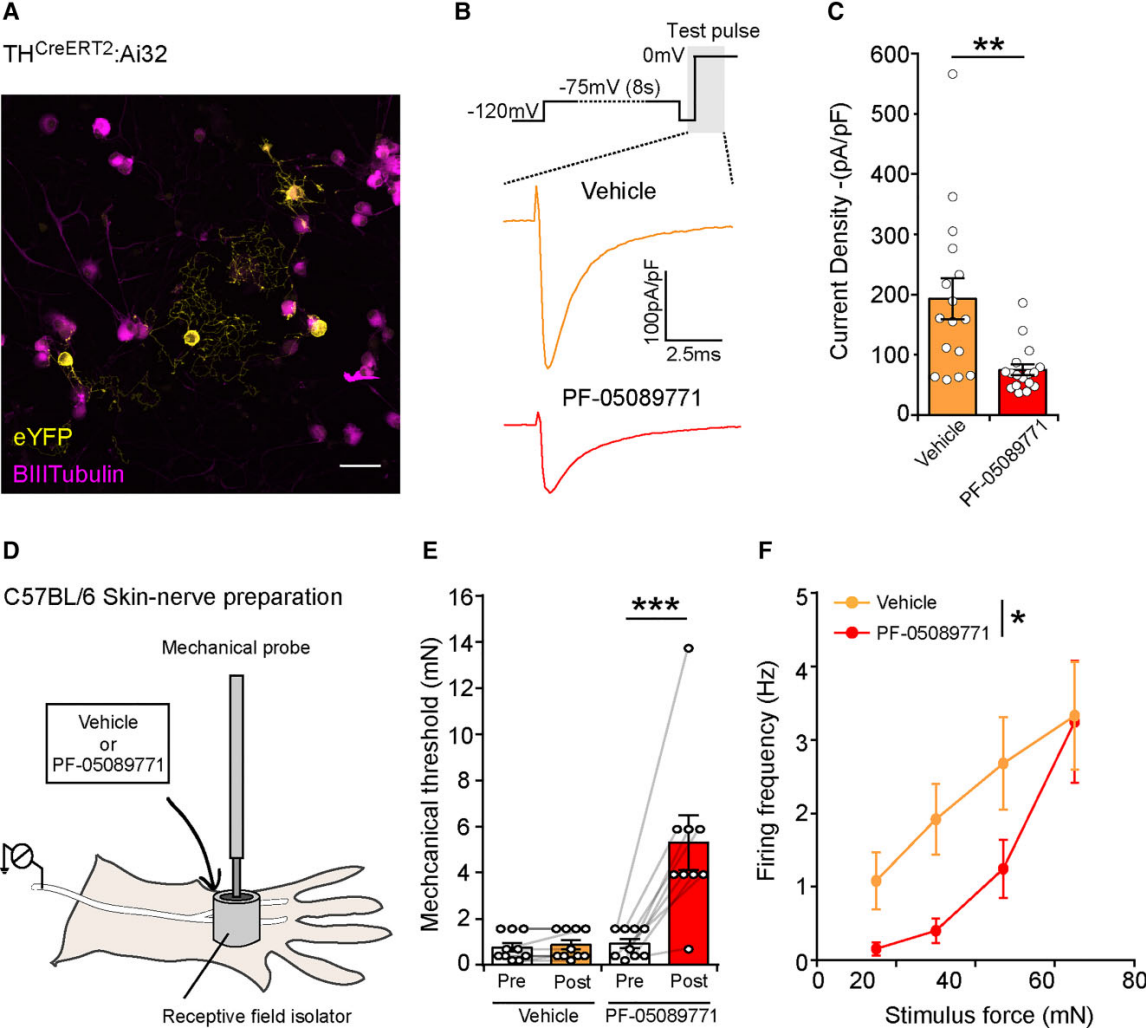
**Selective small molecule inhibition of Na_v_1.7 currents results in hypo-excitable C-LTMR sensory endings**. (**A**) DRG neuronal cultures were made from genetically labelled C-LTMRs (TH^CreERT2^Ai32/eYFP) and used for subsequent voltage clamp analysis. (**B**) *Top*, During the voltage-clamp protocol, neurons were depolarized to −75 mV from a holding potential of −120 mV for 8 s (to inactivate a proportion of Na_v_1.7 channels), followed by a 2 ms recovery step to −120 mV and a test pulse to 0 mV. *Bottom*, Example traces of total sodium currents induced during the test pulse (shaded region of protocol schematic) in the presence of vehicle or PF-05089771 (10 nM). (**C**) Quantification of the sodium current densities in wildtype C-LTMRs in the presence of vehicle or PF-05089771. Blockage of Na_v_1.7 using PF-05089771 in C-LTMRs results in a significant reduction in the sodium current density compared to vehicle treated C-LTMRs (Vehicle: *n* = 16 cells, PF-05089771: *n* = 17 cells, Mann–Whitney U-test, U = 49, *P* = 0.0012, **) (**D**) Example illustration of single-fibre C-LTMR recordings from WT mice. C-LTMRs were identified and subsequently recorded following a 10 min incubation of vehicle or PF-05089771 (10 nM) applied directly to the isolated receptive field. (**E**) C-LTMR mechanical thresholds pre and post vehicle or PF-05089771. Small molecule inhibition of Na_v_1.7 in C-LTMR sensory endings results in a significant increase in the mechanical threshold compared to vehicle. [Vehicle: *n* = 10 units, PF-05089771: *n* = 9 units, repeated measures two-way ANOVA *F*(1, 17) = 12.66, *P* = 0.0024, **, with Bonferroni *post hoc* tests, vehicle pre versus post: *t* = 0.151, *P* = > 0.99, n.s, PF-05089771 pre versus post, *t* = 5.82, *P* = <0.0001, ***]. (**F**) The increasing force stimulus-response function showing that PF-05089771 treated C-LTMRs are significantly hypo-excitable to supra-threshold stimuli compared to vehicle treated C-LTMRs [Vehicle *n* = 10 units, PF-05089771 *n* = 9 units, two-way ANOVA, *F*(1, 68) = 6.95, *P* = 0.0104, *]. All data represented as mean ± SEM. *P < 0.05, **P < 0.01, ***P < 0.001.

The small molecule inhibitor PF-05089771 shows selectivity for Na_v_1.7^39^ and has been in clinical development; it has shown potential in the treatment of patients with inherited erythromelalgia.^30^ We performed voltage clamp recordings of labelled C-LTMRs (Fig. 6A) to investigate the effects of PF-05089771 (10 nM) on sodium current densities. Small molecule blockade of Na_v_1.7 significantly reduced the total sodium current density in C-LTMRs (Fig. 6B and C). We next performed primary afferent recordings using the *ex vivo* skin-nerve preparation from WT mice in the presence or absence of PF-05089771 to determine whether this selective small molecule blocker could impact rodent C-LTMR function. We identified and isolated C-LTMR receptive fields in rodent hairy skin and applied either vehicle or PF-05089771 and assessed excitability (Fig. 6D). We compared the mechanical thresholds of the isolated C-LTMR receptive fields before and after treatment and discovered that, compared to vehicle, there is a significant increase in mechanical thresholds when PF-05089771 is applied (Fig. 6E). In addition, when we assessed the stimulus response function using suprathreshold mechanical stimuli, we observed a significant reduction in C-LTMR activity in the presence of PF-05089771 compared with the vehicle (Fig. 6F). To summarize, selective small molecule blockade of Na_v_1.7 reduced total sodium currents and altered C-LTMR function, resulting in hypo-excitability.

#### Computational modelling of human *SCN9A* mutations in C-LTMRs

**Figure 7:**
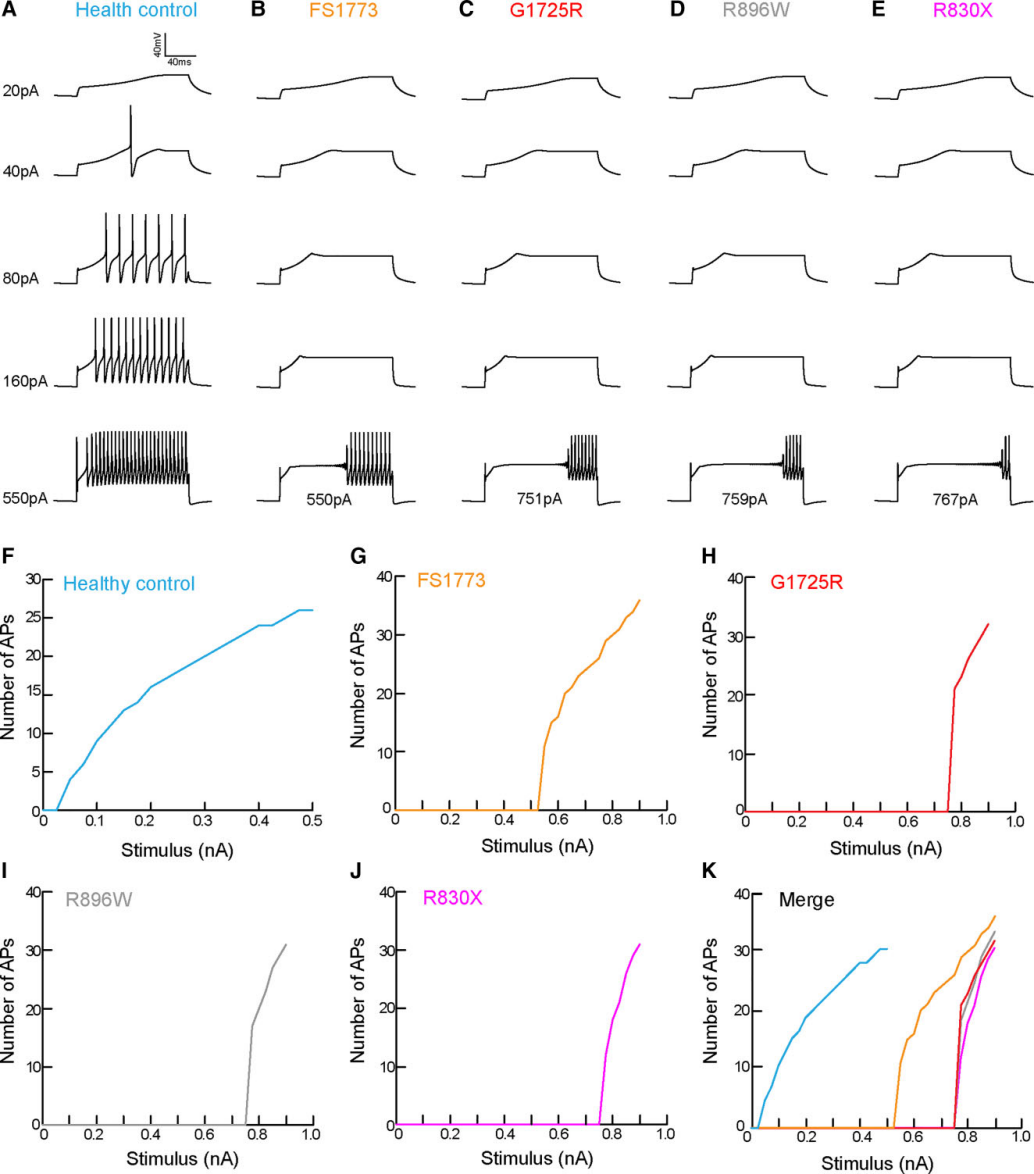
**Modelling of human SCN9A mutations in a C-LTMR computational model shows hypo-excitability**. (**A**) Computational modelling of healthy control participant C-LTMRs with no mutations in Na_v_1.7. Example traces of C-LTMR excitability and firing patterns assessed by increasing the current injected into the model. Examples of C-LTMR excitability and firing patterns when the model was adapted to take into account the changes in Na_v_1.7 conductance due to the following SCN9A mutations: (**B**) FS1773; (**C**) G1725R; (**D**) R896W; and (**E**) R830X. The overlaid current value denotes the threshold of each model. All SCN9A mutations have increased thresholds to current injections. (**F**) The healthy control model was subsequently run to increase the current injection successively by 25 pA in order to assess suprathreshold excitability. The model was adapted (change in Na_v_1.7 conductance) to take into account each SCN9A mutation [(**G**) FS1773; (**H**) G1725R; (**I**) R896W; and (**J**) R830X] and executed to assess suprathreshold excitability. (**K**) The merge of all models clearly illustrates that all SCN9A mutation models are hypo-excitable and require much larger current injections in order to repetitively fire to the same frequency as the healthy control C-LTMR model.

Given the clinical data illustrating that CIP participants experience an altered affective touch perception and that genetic ablation or pharmacological blockade of Na_v_1.7 in the rodents reduced C-LTMR excitability, we investigated the impact of the CIP participant mutations on C-LTMR excitability and function. To address this question, we took advantage of the recent Na_v_1.7 mutation characterization from three CIP participants in our cohort.^26^ From the data available in McDermott *et al.*^26^ we were able to calculate the fold decrease in Na_v_1.7 conductance as a consequence of each *SCN9A* mutation (Supplementary Table 2). We used a publicly available computational model of C-LTMR sensory neurons^17^ to model C-LTMR excitability, while altering the Na_v_1.7 conductance (Na_v_1.7 g-CLTMR, mS/cm^2^) in accordance with the conductance decrease observed in each CIP participant mutation (Supplementary Table 2). We ran the model in the naïve setting, without changing the Na_v_1.7 conductance, in order to resemble healthy control participant excitability measures (Fig. 7A). Next, we ran the model for four mutations from three CIP participants using the new Na_v_1.7 conductance values calculated (Fig. 7B–E). C-LTMR excitability was strikingly impaired when modelling CIP participant mutations in C-LTMRs (Fig. 7B–E). The minimum current required to elicit an action potential in healthy control models was 40 pA; however, in CIP mutation models this ranged from 550 pA–767 pA, depending on the mutation (Fig. 7A–E). We also used these computational models to look at suprathreshold excitability. We modelled C-LTMRs receiving incremental current injections (Δ25 pA) and there was clear hypo-excitability observed in all CIP mutation models compared with the healthy control model (Fig. 7F–K).

## Discussion

We have found that not only do humans with *SCN9A*-LOF mutations have CIP but also an altered experience and perception of affective touch sensation. We demonstrated that rodent C-LTMRs express high levels of Na_v_1.7 and used selective genetic strategies to attribute the LOF phenotype to hypo-excitable C-LTMR primary afferents. We also challenged the current concept that pharmacological/genetic blockade of Na_v_1.7 is selective to the nocioceptive system and showed that the affective touch system, cool sensitivity and mechanical low-force discrimination also requires functional Na_v_1.7.

The CIP participants in this study were all bi-allelic compound heterozygotes, and all of their mutations have previously been characterized and shown to abolish almost all Na_v_1.7-driven sodium currents. The CIP participants have also been studied extensively in the context of nocioception.^18,24^ These participants have never experienced pain and as a result have had multiple injuries throughout their lives due to a loss of functional C-nocioceptors, a lack of epidermal small fibres^26^ and up-regulation of endogenous opioids.^28^ These patients also lack itch perception in response to pruritogens, such as histamine, and demonstrate mild hyposensitivity to warm and cool stimuli, which we have re-examined in this study. Vibration and mechanical detection thresholds have not been found to differ from control individuals; however, we also re-examined low-force punctate monofilament discrimination in this study using a sensitive measure of light touch discrimination. The affective touch system has not previously been investigated in these patients.

The identification of human mutations affecting C-LTMRs and affective touch is scarce. Patients with hereditary sensory and autonomic neuropathy type-III (HSAN-III) and type-V (HSAN-V), who also have congenital insensitivity to pain, have a reduced affective touch percept.^50,51^ HSAN-V is due to a mutation in the nerve growth factor beta gene (*NGFβ*). NGF mediates its effects by binding to the NTRK1 receptor; the NTRK1^+^ lineage of small-diameter, unmyelinated neurons derive from the *Neurog1* wave of neurogenesis, and these mutations likely impede the initial development of C-LTMRs.^52^ We have now shown that LOF mutations in the voltage-gated ion channel Na_v_1.7 have negative impacts on affective touch. Our cohort of CIP participants exhibited altered self-reported pleasantness for gentle dynamic brush stimulation and did not show the classical inverted U-shaped response pattern. Compared with a less pleasant fast touch stimulus, affective touch reliably attenuates activity of the corrugator muscle in an unbiased facial EMG measure of affective touch, suggestive of a reduction in negative affect.^36,41^ Here, 3 cm/s stroking stimulation failed to influence corrugator activity in CIP participants, suggesting that touch which normally elicits human C-LTMR activation does not attenuate a negative affect in these individuals. This was selective for the affective aspect of the stimulus, since all CIP participants were able to rate the stimulus intensity similarly to healthy control participants. This confirms previous literature suggesting that in cases of CIP the large myelinated touch fibres are not compromised.^26^ Collectively, these findings demonstrated that human *SCN9A*-LOF mutations can alter the affective component of pleasant touch sensation.

To further investigate the expression and role of Na_v_1.7 in C-LTMR function, we took advantage of rodent models. Na_v_1.7 is known to be expressed in nocioceptors.^44,53^ Using *in situ* hybridization and analysis of previously published data sets,^45^ we showed that *SCN9A* is expressed in all C-LTMRs and to a high level. These data add to the recent sequencing data showing that *SCN9A* is most highly expressed in the rodent C-LTMR population.^17^ Interestingly many A-LTMRs also show some expression of Na_v_1.7; however, there is no evidence of impairments either in large fibre-mediated touch modalities or the electrophysiological properties of these afferents in *SCN9A*-LOF CIP patients.^24–26^ This is likely due to functional redundancy and the co-expression (unlike in C-LTMRs^17^) of other TTX-S VGSCs such as Na_v_ 1.1 and 1.6 in these neurons, which can compensate for the loss of Na_v_1.7.^18^

A number of genetic KO strategies have been used previously to investigate the role of C-LTMRs in rodents. A global vGLUT3 KO, initially thought to be C-LTMR-specific, resulted in altered noxious mechanical thresholds,^10^ a phenotype which was later shown to be driven by loss of spinal vGLUT3.^54^ Other studies suggest deficits in acute light touch, cold detection and chemical pain responses when Na_v_1.8 positive sensory neurons (which include C-LTMRs) lack Cav3.2, a voltage-gated calcium channel enriched in C-LTMRs.^55^ In contrast, the global KO of the chemokine-like protein Tafa4, which is thought to only be expressed and released by C-LTMRs, resulted in a pro-nocioceptive phenotype.^11^ This phenotype was recovered by administration of exogenous Tafa4, a mechanism which involves GABAergic transmission and spinal microglia.^11,56^ Vrontou *et al*.^57^ identified a population of sensory neurons that expressed the G-protein coupled receptor MrgprB4 and provided evidence that this population is involved in massage-like stroking of hairy skin. Unfortunately, Vrontou *et al.*^57^ were not able to identify this population as a low-threshold mechanoreceptive population and could only infer that they are C-fibres due to molecular traits;^57,58^ there has been no physiological evidence of C-fibre range conduction velocities of MrgprB4+ afferents. There is closer alliance and more physiological evidence that the TH/vGLUT3/Tafa4 population is the likely species equivalent of human C-LTMRs.^10,11,13^ However, we cannot exclude the possibility that both populations co-exist and that perhaps relates to modality-specific pleasure perception.

Hitherto, there has been a lack of transgenic tools available to selectively target the C-LTMR population; the discovery that TH is a marker of C-LTMRs and development of the TH^CreERT2^ line has helped delineate the physiology and connectivity of these neurons in the rodent.^13,46^ In validating the TH^CreERT2^ mouse (and confirming that it is a good model system to target C-LTMRs^46^), we have found that C-LTMRs innervate not only hairy skin on the dorsum of the paw but also the hind-paw plantar surface of mice, as longitudinal lanceolate endings. These hair follicles located between the hind-paw running pads were thought to be innervated exclusively by the Aδ-LTMR population known as D-hairs.^47,59^ This is an important finding, as studies often overlook these particular hair follicles, and the dogma currently suggests that C-LTMRs do not innervate rodent plantar skin. We have provided evidence that this population does innervate the running pad region, which is commonly tested in rodent sensory biology.

We saw that when we ablated Na_v_1.7 in rodent C-LTMRs, the response to noxious heat remained intact, but mice became hyposensitive to punctate mechanical stimuli. This finding is consistent with previous rodent studies which also show that C-LTMRs have a modest contribution to punctate mechanical stimuli.^55^ There is debate in the literature over the perceptual correlate of a withdrawal to a von Frey hair in rodents. It is likely that von Frey withdrawal relates to stimulus detection rather than a painful aversion. The genetic ablation of all TRPV1-lineage neurons (all nocioceptors) or the optogenetic silencing of CGRP+ peptidergic neurons did not alter von Frey thresholds in the naïve, uninjured state.^60,61^ In addition, the early human data which first characterized C-LTMRs and human microneurography we present in this study demonstrate that C-LTMRs do respond to both punctate and brush stimuli.^3^ Furthermore, patients lacking A-fibre function are still able to detect low-force punctate monofilaments, but only in hairy skin.^62^ This led us to reassess low-force punctate monofilament discrimination in a single CIP participant using a more sensitive tactile task (more sensitive than quantitative sensory testing). We showed for the first time that human C-LTMRs can encode low indentation forces in healthy participants, and that the ability to discriminate between low force punctate mechanical stimuli was reduced in one of the *SCN9A*-LOF participants. Together this highlights that C-LTMRs play a role in punctate mechanical detection in rodents and humans.

Interestingly, using our mouse KO model, we did not see any changes in the number of responses to dynamic light touch stimuli. However, one must consider that these assays interrogate stimulus detection, not the affective component of the stimulus. Our human data suggested that *SCN9A*-LOF participants do not fail to detect the brush stimuli, but rather it is the affective perception that is altered. The sensory biology field is currently challenged in not having a reliable read-out for affective pleasure sensation in rodents, an obstacle that as a community we need to overcome. The loss of C-LTMR Na_v_1.7 resulted in deficits in cool coding at the behavioural and electrophysiological levels (discussed further below), suggesting that without Na_v_1.7 in the C-LTMR population, mice are unable to code thermal stimuli properly. One idea is that C-LTMR activity contributes to thermal preference, and loss of Na_v_1.7 leads to mice having altered thermal preferences and spending more time in cooler (non-noxious) regions. We have re-examined human data from a previous study, in which quantitative sensory testing was used to sensory profile CIP participants, and it was found that they were hypo-sensitive to cooling stimuli.^26^ There are few other regions of the nervous system that also co-express TH and Na_v_1.7 where ablation would also occur in our model. Sympathetic neurons express both;^15^ however, they unlikely require Na_v_1.7, as CIP participants do not present with sympathetic deficits. Equally, dopaminergic neurons in the periaqueductal grey and ventral midbrain express TH but show very low levels of *SCN9A* expression.^15^ Finally, some populations of jugular/nodose sensory neurons express both TH and Na_v_1.7.^63^ However, it is reported that TH+ jugular sensory neurons can be molecularly classified as C-LTMRs,^63^ and nodose sensory neurons innervate visceral organs, i.e. not regions we have tested in this study.

Given the high expression of Na_v_1.7 in C-LTMRs and the important role of Na_v_1.7 as a threshold channel within sensory neurons, we investigated whether the observed behavioural changes were due to alterations in the excitability of C-LTMRs. Using voltage-clamp recordings *in vitro,* we saw a reduction of the sodium current density in C-LTMRs which lack Na_v_1.7 and found that there is a large contribution of Na_v_1.7 to the total sodium currents in this population. A previous study used the skin-nerve preparation to investigate Na_v_1.7 contribution to peripheral nerve excitability, using a sensory neuron specific Na_v_1.7 KO mouse.^64^ However, Hoffmann *et al.*^64^ only recorded from two C-LTMRs in each genotype, and therefore the study was too underpowered to draw any conclusions as to the stimulus-response function of these units. We therefore assessed C-LTMR primary afferent terminal excitability in detail using the skin-nerve preparation. Here, we found that loss of Na_v_1.7 results in hypo-excitability and, in particular, alters C-LTMR stimulus response functions to static and dynamic punctate mechanical stimuli. In addition, we directly characterized the thermal response of C-LTMRs and observed that they respond to both warming and cooling stimuli with an activity pattern that is consistent with an inverted U-shaped response. Peak activity of this inverted U-shaped response was ∼27–28°C, which is consistent with our thermal gradient behavioural finds, where thermal preference is also ∼28°C in WT mice. The relationship between thermal response of C-LTMRs and subjective preference in mice resonates well with human evidence. In a combined microneurography and psychophysics experiment, Ackerley *et al*.^65^ replicated the typical speed-dependent inverted U-shaped response in C-LTMRs, together with increased C-LTMR-firing to neutral (32°C) compared with warm (42°C) and cool (18°C) stroking temperatures. Importantly, the speed-dependent vigorous response to stroking stimuli at neutral temperatures was positively correlated to self-reported pleasantness, indicating a link between C-LTMR firing properties and subjective preference. Our findings in the mouse also support previous studies that implicate C-LMTRs in thermal preference. TH-positive C-LTMRs are sensitive to cooling stimulation^13^ and an altered thermal preference was observed in mice with hyposensitive C-LTMRs due to lack of the voltage-gated calcium channel Cav3.2.^55^ Additionally, recent work used activatory chemogenetic tools to selectively increase C-LTMR activity, which resulted in increased thermal preference and induced a place preference in mice.^66^ We provide evidence that C-LTMR activity in response to thermal stimuli may underlie thermal preference in mice. These findings are consistent with the idea that C-LTMR activity may underlie social thermoregulation, such as mammalian huddling behaviours, which are important for survival.^67^ Thermal characterization following Na_v_1.7 ablation in C-LTMRs results in hypo-excitability of C-LTMRs to cooling stimuli, a loss of the inverted U-shaped response pattern and a shift in the thermal preference of behaving mice.

From this, we propose a mechanism whereby Na_v_1.7, which has a large contribution to C-LTMR sodium currents, is important in regulating C-LTMR excitability and function. We propose that loss of functional Na_v_1.7 in our CIP participants likely results in hypo-excitable C-LTMRs which can no longer effectively drive the affective component of pleasant brush stimuli, monofilament discrimination and cool sensibility.

Targeting Na_v_1.7 to therapeutically treat painful conditions may therefore have unintended consequences on C-LTMRs and the affective touch system. We indeed saw a reduction in both C-LTMR sodium currents and terminal excitability when using a Na_v_1.7-selective small molecule blocker. These data suggest that current and future strategies, which target Na_v_1.7 to treat pain, need to consider the consequences of reducing the excitability of this non-nocioceptive population and how this might alter social touch, relationships and regulation of stress response.^67^ Whether these are clinically relevant side effects remains unknown.

We know from previous studies that human *SCN9A*-LOF mutations can reduce neuronal excitability.^26,29^ As such, we sought to investigate the effects of human *SCN9A*-LOF mutations in the context of C-LTMRs using a recently developed C-LTMR computational model.^17^ While models of C-nocioceptors exist, there is strong evidence that these would not generalize to C-LTMRs.^68^ For instance, C-LTMR activity-dependent slowing is very different compared with C-nocioceptors.^69,70^ Therefore, using a C-LTMR-specific computational model, we recapitulated mutations from a subset of our CIP participant cohort and, consistent with our empirical findings, these led to hypo-excitability in C-LTMRs. The CIP participants had compound heterozygote mutations, so the outcome *in vivo* was the combinatorial effect of two mutations.

To summarize, we used a multidisciplinary approach to investigate the role of Na_v_1.7 in C-LTMR function in humans and mice. Psychophysical testing showed CIP participants have an altered perception of affective touch sensation, deficits in low-force monofilament discrimination and cool sensibility. We used a mouse model to selectively ablate Na_v_1.7 in C-LTMRs in order to determine this mechanism. We found that loss of Na_v_1.7 in C-LTMRs resulted in behavioural hyposensitivity to punctate mechanical stimuli, deficits in cool sensibility and an altered thermal preference. Loss of Na_v_1.7 in C-LTMRs resulted in a reduction in sodium currents and hypo-excitability to mechanical and cooling stimuli. Pharmacological blockade of Na_v_1.7 also led to hypo-excitable C-LTMRs. The impact of loss of function in one VGSC alpha subunit within different sensory neuron sub-populations is dependent on co-expression with other VGSCs, which vary between fibre types, and the non-redundant role of Na_v_1.7 in C-LTMRs which we observed, is supported by a recent computational model of C-LTMRs. The phenotype of bi-allelic LOF gene mutations in *SCN9A* has therefore been widened to not only include pain perception but also impaired pleasant touch perception. Furthermore, targeting Na_v_1.7 to therapeutically treat painful conditions may have implications on the affective touch system.

## Supplementary Material

awab482_Supplementary_Data

## Abbreviations

C-LTMR: C-low threshold mechanoreceptor
CIP: congenital insensitivity to pain
DRG: dorsal root ganglion
GOF: gain-of-function
KO: knock-out
LOF: loss-of-function
TH: tyrosine hydroxylase
VGSC: voltage-gated sodium channel
