# Supplementary material for: Nav1.7 is required for normal C-low threshold mechanoreceptor function in humans and mice
Source: Brain. 2021 Dec 27;145(10):3637–53. doi: 10.1093/brain/awab482 (PMC9586547; doi:10.1093/brain/awab482)
Supplement: awab482_Supplementary_Data [file awab482_supplementary_data.pdf]

Supplementary Materials for

Nav1.7 is required for normal C-Low threshold mechanoreceptor function in humans and mice

Steven J. Middleton,[1,†] Irene Perini,[2,3†] Andreas C. Themistocleous,[1,4] Greg A. Weir,[1,5] Kirsty McCann,[1] Allison M. Barry,[1] Andrew Marshall,[6] Michael Lee,[7] Leah M. Mayo,[2] Manon Bohic,[8,9] Georgios Baskozos,[1] India Morrison,[2] Line S. Löken,[10] Sarah McIntyre,[2] Saad S. Nagi,[2] Roland Staud,[11] Isac Sehlstedt,[12] Richard D. Johnson,[10,11] Johan Wessberg,[10], John N. Wood,[13] Christopher G. Woods,[14] Aziz Moqrich,[8] Håkan Olausson,[2,‡] and David L. Bennett[1,‡]

[†, ‡] These authors contributed equally to this work.

Correspondence to:

David L. Bennett (david.bennett@ndcn.ox.ac.uk)
University of Oxford
West Wing, Level 6,
John Radcliffe Hospital, Oxford, OX3 9DU
United Kingdom

Håkan Olausson (hakan.olausson@liu.se)
Linköping University
Tinnerbäckshuset, Level 14,
58185, Linköping
Sweden

This PDF file includes:
Supplementary Figures 1 to 7
Supplementary Tables 1 to 3
Supplementary Methods

Supplementary Figures

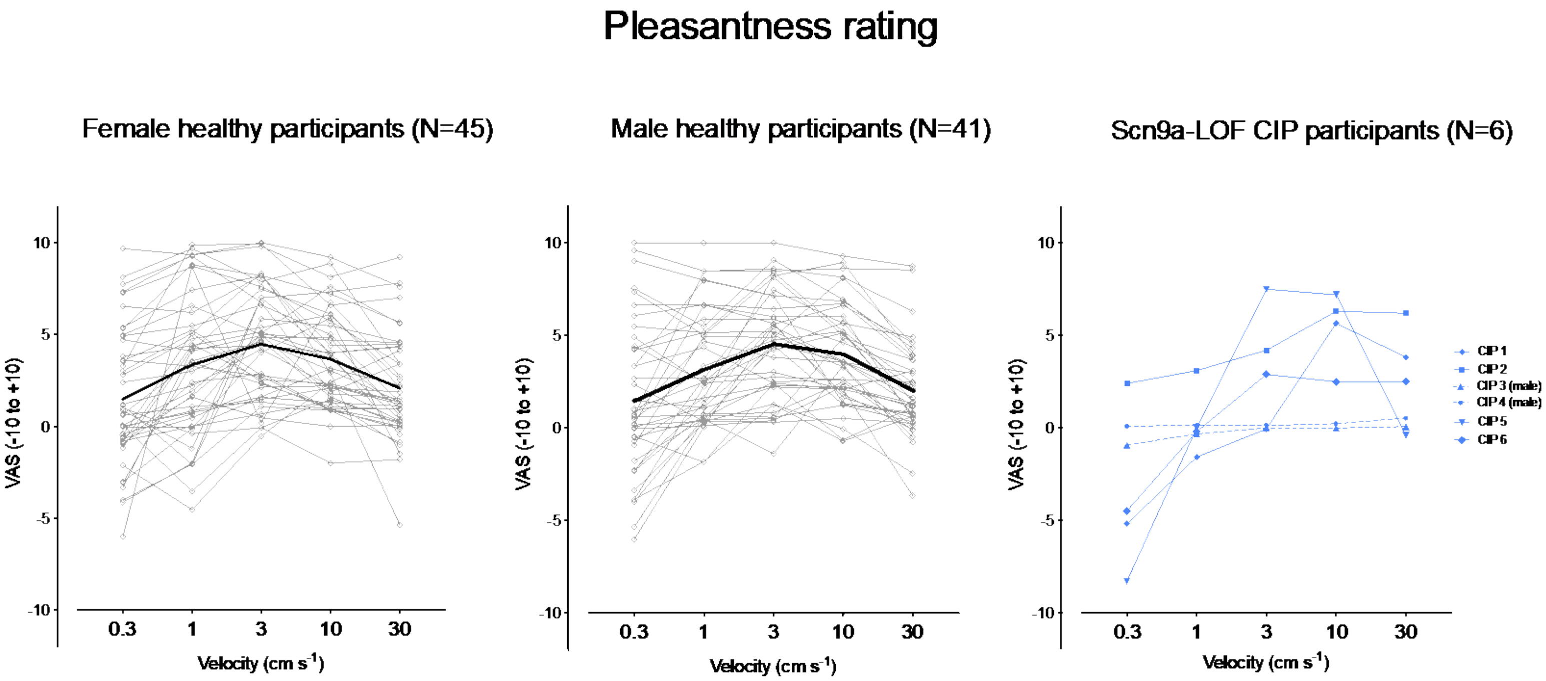

Supplementary Figure 1. Individual pleasantness VAS ratings. Individual touch pleasantness ratings across five stroking velocities in female and male healthy participants (black lines reflect mean values) and in CIP participants reported in Figure 1. In healthy participants (n = 86), the 2x5 ANOVA with factors speed (0.3, 1, 3, 10, 30 cm s^{-1}) and sex (female, male) revealed no between-group effect nor a speed by group interaction (Ps > 0.8).

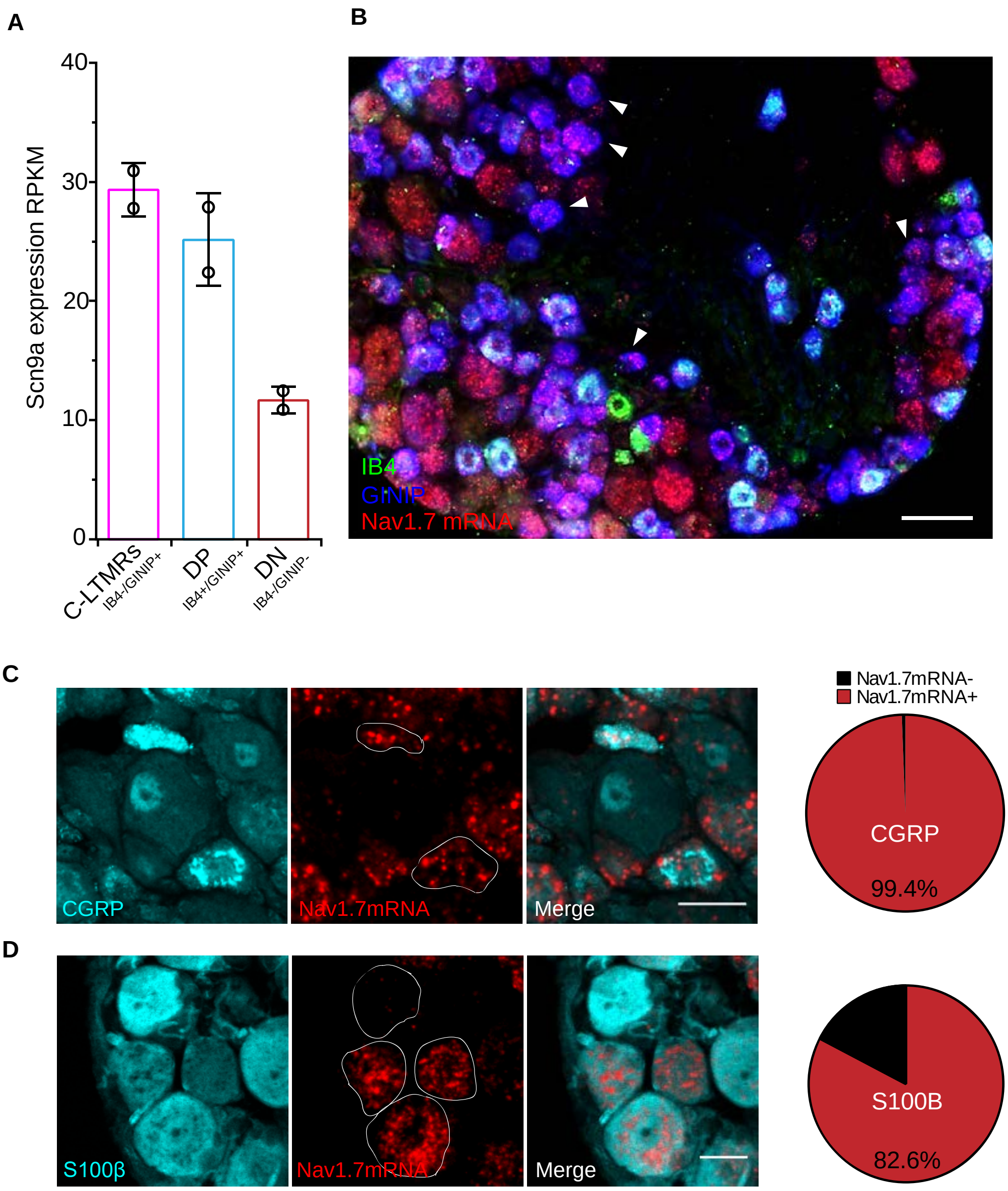

Supplementary Figure 2. SCN9A mRNA expression in IB4-GINIP+ C-LTMRs and other sensory neuron populations. (**A**) Relative gene expression for SCN9A was quantified by the normalised reads per kilobase per million mapped reads (RPKM) using the mean and the standard deviation across three distinct neuronal populations, C-LTMRs, double positive non-peptidergic nociceptors (IB4+GINIP+) and double negative all other neurons (IB4-GINIP-). RPKM data were downloaded from GSE64091. Data was originally presented in Reynders *et al* (2015). *n* =3 per respective neuronal subpopulation. (**B**) Validation of the sequencing data shown by ISH of Nav1.7mRNA and IHC co-localisation of IB4 and GINIP in wild type DRG sections. Example C-LTMRs shown with arrows. Scale bar 50um. (**C**) ISH of DRG sections showing the percentage co-localisation of CGRP positive (peptidergic) nociceptors and

Nav1.7mRNA. Scale bars 25µm. (**D**) Percent co-localisation of S100β positive (myelinated) afferents and Nav1.7mRNA. Scale bars 25µm.

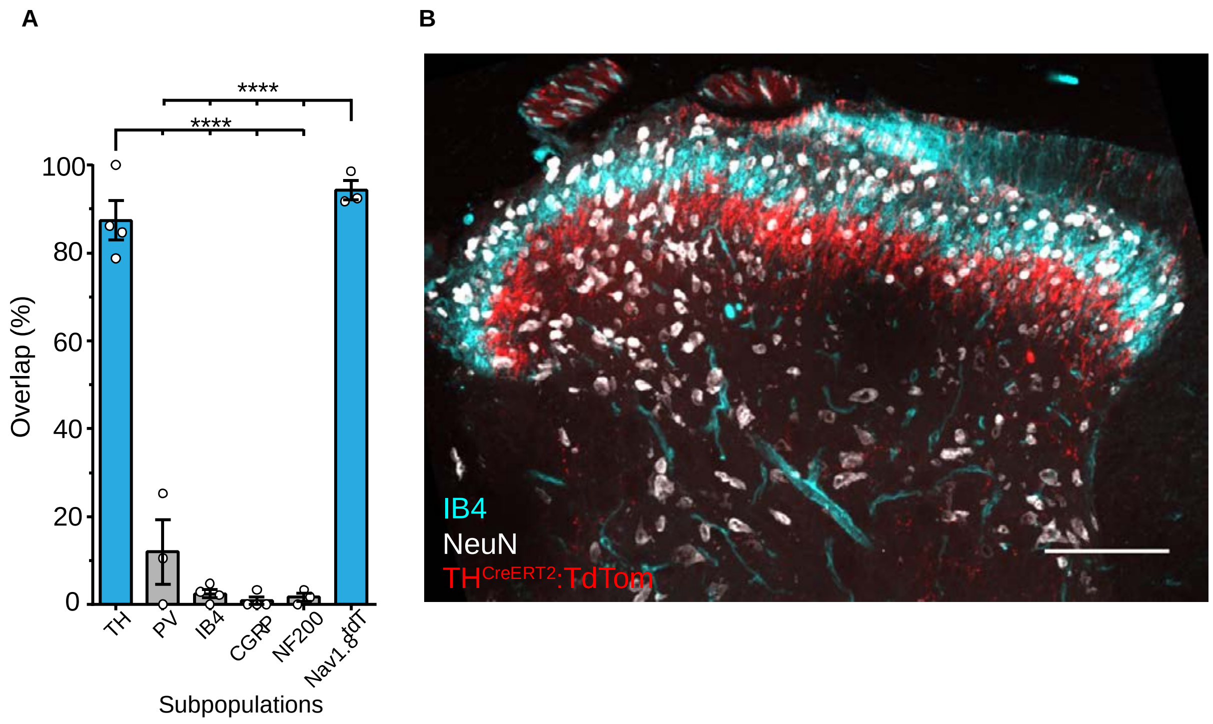

Supplementary Figure 3. TH^{CreERT2}tdTom histological characterization. (**A**) Quantification of the TH^{CreERT2}tdTom co-localisation with other sensory neuron markers as described in Fig.3. There is significant co-localisation of TH antibody/TH^{CreERT2}TdTom and TH antibody/Nav1.8^{Cre}tdTom cells compared to each other population marker (n = 3 or 4 mice, overlap averaged from 3 sections per mouse. One-way ANOVA, F = 171.0, P < 0.0001, Bonferroni post hoc test, P < 0.0001, ****). All data represented as mean±SEM. (**B**) Dorsal horn of the spinal Cord showing C-LTMR termination in Lamina II^{I} below IB4 positive afferents which terminate in Lamina II°. Scale bar 100µm.

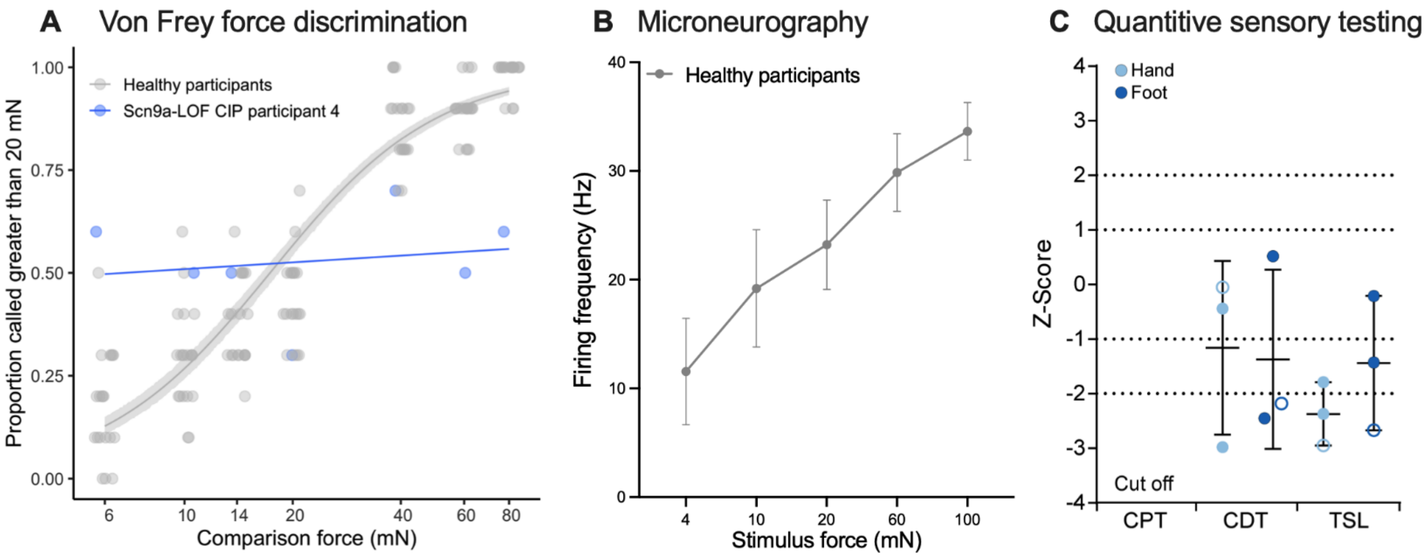

Supplementary Figure 4. SCN9A LOF participants show reduced punctate mechanical discrimination and cooling detection abnormalities. (**A**) The ability to discriminate between low force von Frey monofilaments is reduced in Scn9a-LOF CIP participant 4. Healthy participants (*n* = 20) were stimulated with pairs of monofilament indentations of varying forces, applied for approximately 1 second to the dorsum of the foot. The two stimuli in a pair were presented approximately 3 seconds apart, and the participant was required to judge which one was greater. In each pair, one of the stimuli produced a 20 mN force and the other produced one of seven comparison forces (6 – 80 mN). Each of the 7 comparisons were made 20 times by each participant, in a random order. The experimenter was guided by a computer, which also recorded the participants' responses. The data were analyzed by fitting a logistic regression function to the log of the comparison forces and the corresponding participant judgments. Discrimination capacity is given by the slope of the curve. The control group had a slope of β = 6.23 (95% *CI* = 5.28, 7.70; parametric bootstrap with 1000 samples), while the slope of Scn9a-LOF CIP participant 4 was much shallower (β = 1.1, 95% *CI* = 0.650, 1.94), indicating poorer discrimination of monofilament forces. The experiment control script and analysis script are available (https://github.com/SDAMcIntyre/Expt_MonofilamentDiscrimination). (**B**) Human C-tactile fibers can encode low indentation forces. Single-unit microneurography recordings were performed from the radial nerve of healthy participants (*n* = 5, 18 to 30 years, 3 females) to determine the responsiveness of C-tactile fibers to skin indentations applied manually using von Frey filaments. The mean (± SEM) firing frequency of five C-tactile fibers to indentation onset (500 ms) is shown. A significant linear fit was displayed (R^2 = 0.84, P =

0.03). The C-tactile fibers were located on the distal forearm and the radial hand. They were sensitive to soft brush stroking and had mechanical thresholds ≤ 1.6 mN (median = 0.7, Q = 0.1–1.2). Data collection and analysis procedures were as described previously.[1] **(C)** Quantitative sensory testing (QST) in 3 CIP participants (CIP participants 2, 3, 4) expressed as a z-score using the German neuropathic pain consortium protocol.[2] CPT- cold pain threshold, CDT- cold detection threshold, TSL – thermal sensory limen, Cut off – patients reached cut off without reporting pain. CIP patients display cool/cold sensory hyposensitivity. There is hyposensitivity (a negative z-score) in relation to cool detection. The normative range for healthy people is between 2 and -2. Mean ± SD. Cool/cold QST data previously published open access in McDermott *et al* 2019.[3]

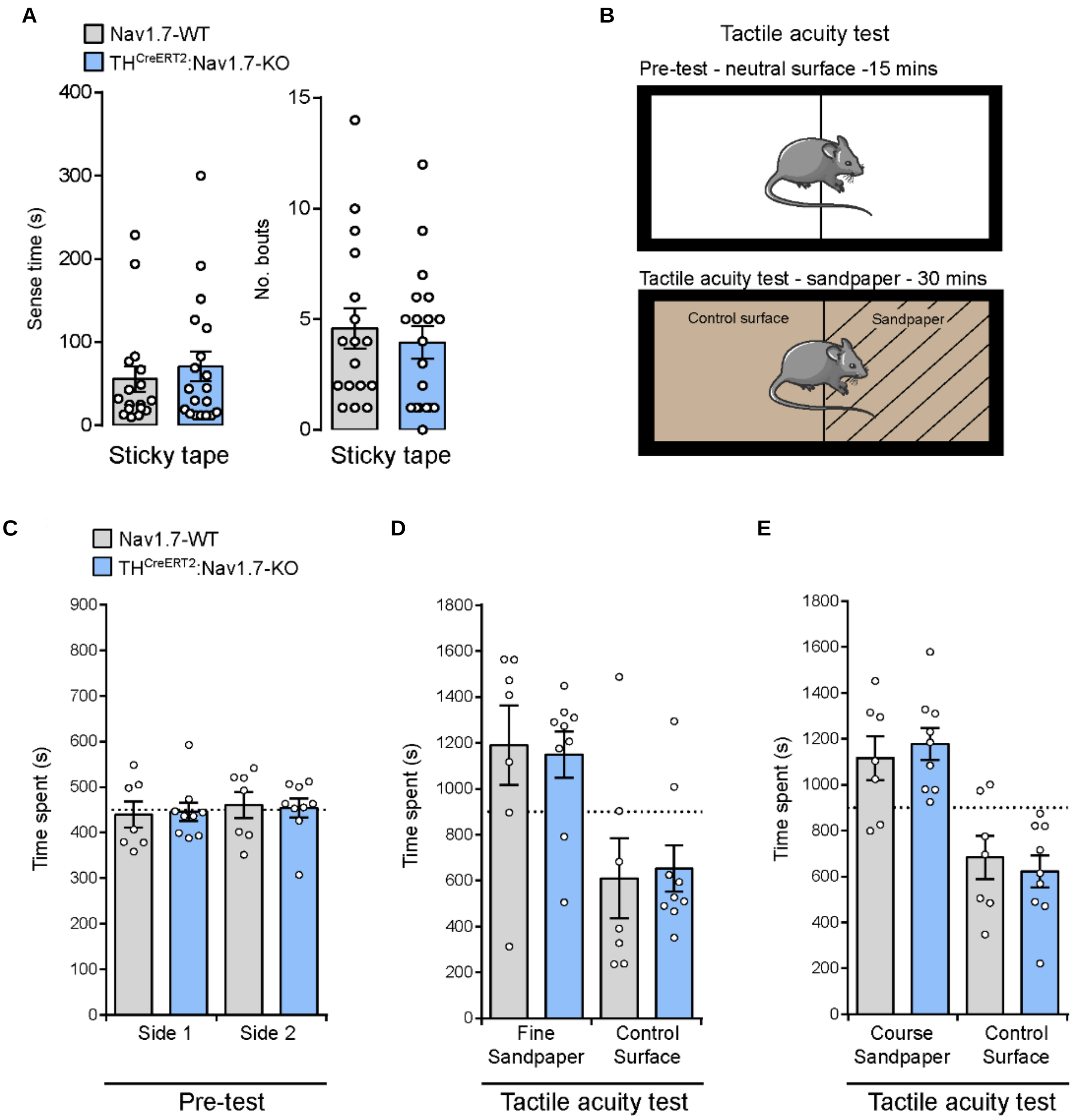

Supplementary Figure 5. Light-touch behaviours in wildtype and THCreERT2:Nav1.7-KO mice. (**A**) The sense time and no. bouts analysed during the sticky tape assay. The sense time and no. bouts were similar for both groups. (Sense time: Mann Whitney U test, $U = 152$, $P = 0.771$. No. Bouts: Mann Whitney U test, $U = 145.5$, $P = 0.617$). (**B**) Illustrative example of the tactile acuity sandpaper assay. (**C**) During pre-testing, mice from both groups spent time equally between side 1 and side 2. Mice from both groups spent more time on the fine (**D**) and course (**E**) sandpaper compared to the control surface. However no significant difference in time spent was observed between groups (Two-way ANOVA, Bonferroni post-hoc, fine sandpaper WT, 7 mice vs KO, 9 mice: $t = 0.22$, $P = 0.99$. Course sandpaper WT, 7 mice vs KO, 9 mice: $t = 0.53$, $P = 0.99$).

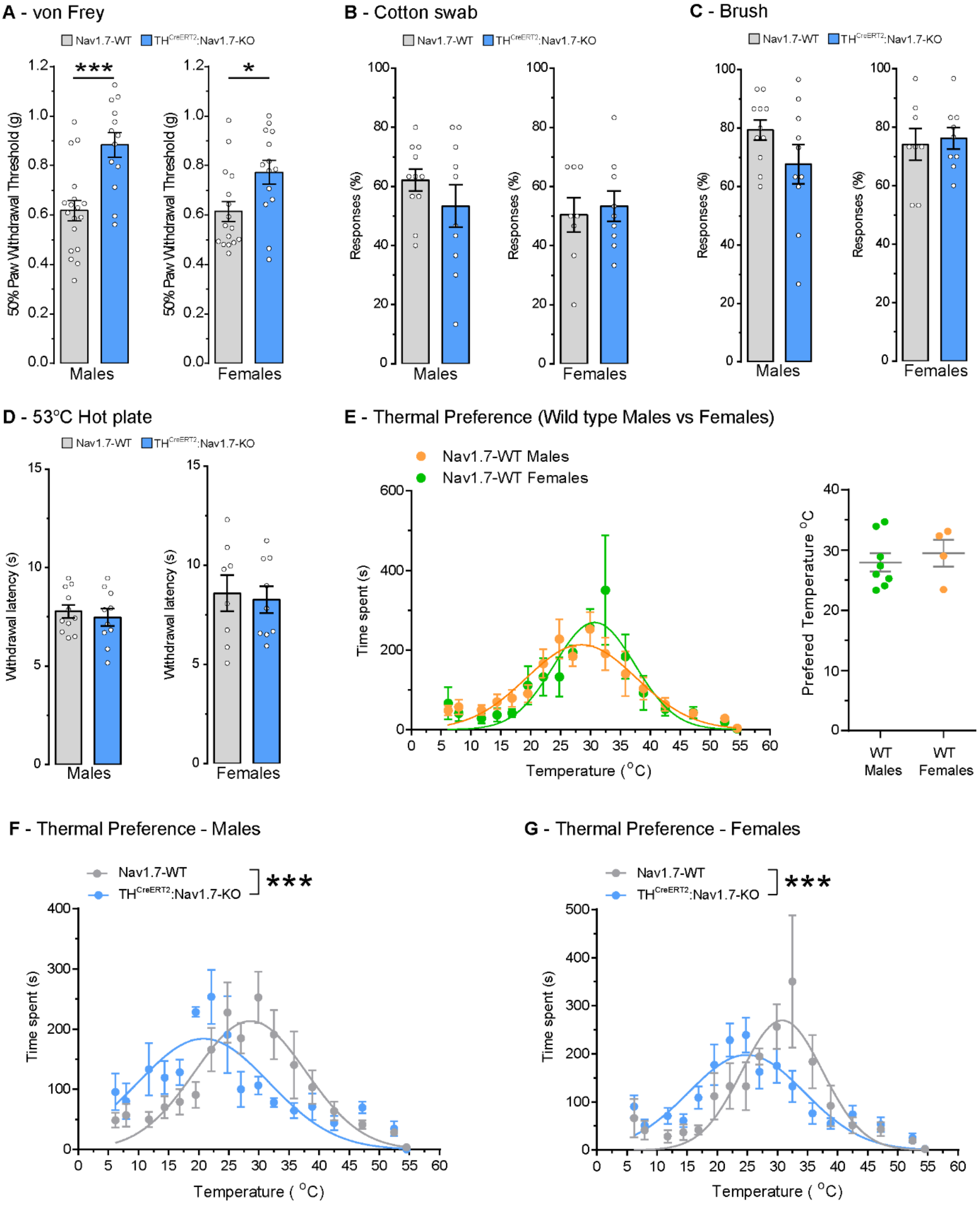

Supplementary Figure 6. The behavioural consequence of ablating Nav1.7 in C-LTMRs is not sexually dimorphic. (**A**) Both male and female TH^{CreERT2}:Nav1.7-KO mice are hyposensitive to punctate von Frey stimuli (Males: WT *n* = 18 mice, KO *n* = 13 mice, two-tailed Student's unpaired t-Test, $t(29) = 4.125$, $P < 0.0003$, ***. Females: WT *n* = 16 mice, KO *n* = 14 mice, two-tailed Student's unpaired t-Test, $t(28) = 2.539$, $P = 0.017$, *). (**B**) Cotton

swab, (**C**) brush and (**D**) hotplate behaviours are not significantly different between genotypes in both male and female mice. (Cotton swab males: WT n = 11 mice, KO n = 10 mice, two-tailed Student's unpaired t-Test, $t(19) = 1.11$, $P = 0.279$, n.s. Cotton swab females: WT n = 8 mice, KO n = 9 mice, two-tailed Student's unpaired t-Test, $t(15) = 0.375$, $P = 0.375$, n.s. Brush males: WT n = 11 mice, KO n = 10 mice, two-tailed Student's unpaired t-Test, $t(19) = 1.59$, $P = 0.126$, n.s. Brush females: WT n = 8 mice, KO n = 9 mice, two-tailed Student's unpaired t-Test, $t(15) = 0.334$, $P = 0.743$, n.s. 53°C hot plate males: WT n = 11 mice, KO n = 10 mice, two-tailed Student's unpaired t-Test, $t(19) = 0.565$, $P = 0.578$, n.s. 53°C hot plate females: WT n = 8 mice, KO n = 9 mice, two-tailed Student's unpaired t-Test, $t(15) = 0.289$, $P = 0.776$, n.s.). (**E**) The thermal preference test of wild type male and female mice. The Gaussian non-linear regression fitted curve for each data set is displayed. Both male (**F**) and female (**G**) $TH^{CreERT2}$:Nav1.7-KO mice show altered thermal preference behaviours and a leftward shift in the Gaussian non-linear regression fitted curve. (Males: WT n = 8 mice, KO n = 3 mice, non-linear regression F-Test, $F(3, 181) = 8.366$, $P < 0.0001$, ***. Females: WT n = 4 mice, KO n = 6 mice, non-linear regression F-Test, $F(3, 164) = 9.337$, $P < 0.0001$, ***).

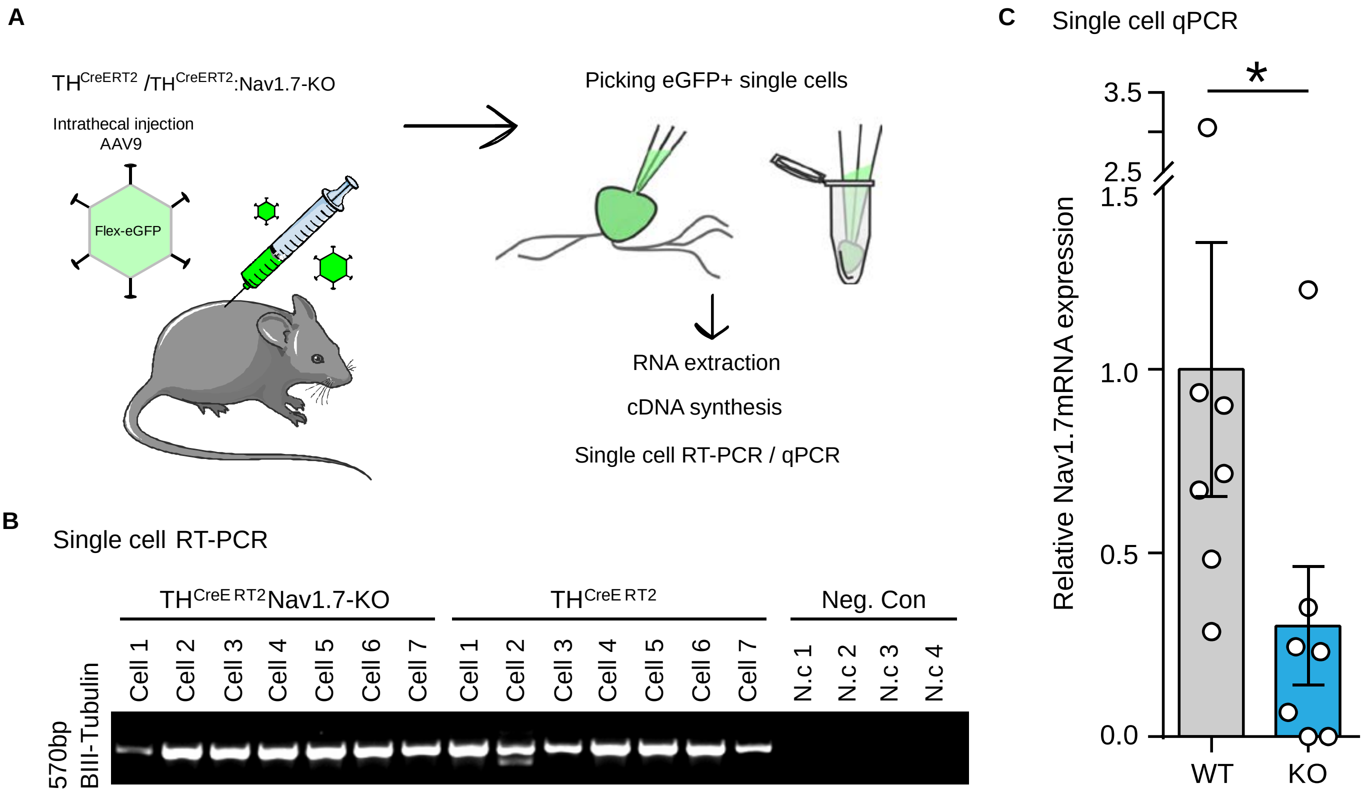

Supplementary Figure 7. Confirmation of the TH^{CreERT2}Nav1.7-KO. (**A**) TH^{CreERT2} (control) or TH^{CreERT2}Nav1.7$^{flox/flox}$ (TH^{CreERT2}Nav1.7-KO) mice received and intrathecal injection of AAV.Flex.eGFP to target C-LTMRs prior to tamoxifen administration. Subsequent tamoxifen injection initiated simultaneous eGFP and Na$_V$1.7 ablation. Virally targeted C-LTMRs were cultured, eGFP expression used to identify the population and single cells "picked", followed by RNA extraction, cDNA synthesis and single cell RT-PCR and single cell qPCR. (**B**) Single Cell RT-PCR for BIII-tubulin was performed on collected samples to confirm that each sample contained cellular RNA, negative controls were clean from contamination. (**C**) qPCR was ran on each single cell sample to assess Nav1.7mRNA expression. There was a significant reduction in Nav1.7mRNA in the TH^{CreERT2}Nav1.7-KO compared to TH^{CreERT2} control. (TH^{CreERT2} n=7 cells, TH^{CreERT2}Nav1.7-KO n=7 cells. Mann Whitney U test, $U = 7$, $P = 0.0256$, *)

Supplementary Tables

Supplementary Table I Demographics and the relevant Scn9a-LOF mutations of the recruited CIP cohort

Participant identifier	Age	Sex	Mutation	Reference
**CIP Participant 1**	16	F	Compound heterozygous mutations: c.5155 T>C; C1719R – in exon 26 causing an amino acid substitution of arginine for cysteine (c.5155 T>C; C1719R) c.3467+3 delA, or IVS17+3delA – in intron 17. This deletion alters the splice donor consensus sequence, which causes exon 17 to be skipped at the mRNA level.	(Staud *et al.*, 2011)[4]
**CIP Participant 2**	44/47[a]	F	Compound heterozygous mutations: c.2691G>A (Y897X) – premature stop codon c.5173G>C (G1725R) – in exon 27, affects a highly conserved region of the protein and is predicted to cause major alteration in the sixth transmembrane region of the protein	(Bogdanova-Mihaylova *et al.*, 2015)[5] (McDermott *et al.,* 2019)[3]
**CIP Participant 3**	34/36[a]	M	Compound heterozygous mutations: c.377+5C>T – intronic variant c.2686C>T (R896W) – in exon 16, affects a highly conserved region of the protein, and is predicted to cause an alteration in the ion transport region of the protein	(McDermott *et al.*, 2019)[3]
**CIP Participant 4**	31/35[a,b]	M	Compound heterozygous mutations: c.2488C>T (R830X) - premature stop codon in coding exon 15 c.5318delA (FS1773) - in exon 26,1 bp deletion that induces a frameshift at position 1773 in the C terminal domain of the channel.	(Ramirez *et al.*, 2014)[6] (McDermott *et al.*, 2019)[3] (Weiss *et al.*, 2011)[7]
**CIP Participant 5**	42	F	Compound heterozygous mutations: Exon 22: c.3699_3709delATGGATAGCAT p.Ile1235LeufsX and in exon 29: c.4975A>T p.Lys1659X. As both of these exons are not last or penultimate, nonsense mediated decay would	(Weiss *et al.*, 2011)[7]

			be predicted for both mutations. *Patient 5 and 6 are siblings	
**CIP Participant 6**	45	F	Compound heterozygous mutations: Exon 22: c.3699_3709delATGGATAGCAT p.Ile1235LeufsX and in exon 29: c.4975A>T p.Lys1659X. As both of these exons are not last or penultimate, nonsense mediated decay would be predicted for both mutations. *Patient 5 and 6 are siblings	(Weiss *et al.*, 2011)[7]

[a]Age during facial EMG testing.

[b]Age during force discrimination testing.

Supplementary Table II CIP participants, their mutations and subsequent changes in Nav1.7 conductance applied to the C-LTMR computational model. Calculated from mutation characterisation in McDermott *et al* (2019).

Participant identifier	*SCN9A* mutation	Fold Decrease in Conductance	gNav1.7-C-LTMR (mS/cm^2)
**Healthy control**	-	-	30.00
**CIP participant 4**	R839X	7.62	3.99
**CIP participant 2**	G1725R	30.20	1.01
**CIP participant 3**	R896W	62.39	0.49

Supplementary Table III List of primary and secondary antibodies

Antibodies	Source	Identifier
**Primary Antibodies**		
NeuN (1:500, Rabbit)	Abcam	Ab177487
NeuN (1:500, Chicken)	Merck Millipore	Abn91
dsRed (1:1000, Rabbit)	Clone tech	632392
Tyrosine Hydroxylase (1:250, Sheep)	Merck Millipore	Ab1542
βIII-Tubulin (1:500, Mouse)	R&D	Mab1195
IB4 (1:50, Streptavidin conjugated)	Sigma-Aldrich	L2140
IB4-Alexa Fluor 488 dye (1:200)	Invitrogen	I21411
GFP (1:500, Chicken)	Abcam	Ab13970
GFP (1:500, Rabbit)	Invitrogen	A6455
PGP9.5 (1:200, Rabbit)	Zytomed	516-3344
CGRP (1:250, Sheep)	Enzo	Ca1137
Parvalbumin (1:200, Guinea pig)	Frontier Institute	Af1000
GINIP (1:1000, Rat)	(Gaillard *et al* 2014)	(Gaillard *et al* 2014)
NF200 (1,1000 Rabbit)	Merck Millipore	ABN76
S100β (1:500, Rabbit)	Abcam	Ab41548
**Secondary Antibodies**		
Alexa Fluor 488 (1:500)	Thermo Fischer Scientific	Alexa Fluor
Alexa Fluor 546 (1:500)	Thermo Fischer Scientific	Alexa Fluor
Alexa Fluor 647 (1:500)	Invitrogen	Alexa Fluor

Pacific Blue (1:100)	Thermo Fischer Scientific	Alexa Fluor
Cy3 (1:500)	Jackson	713-166-147

Supplementary Methods

Humans

Psychophysical correlates to touch were collected in one participant in Gainesville, Florida, USA (participant 1), three participants at the John Radcliffe Hospital, Oxford, United Kingdom (participants 2-4) and two participants at Addenbrook's Hospital, Cambridge, United Kingdom (participants 5 and 6). CIP participants and healthy control participants were examined following the same paradigm. All centres had been trained in carrying out the brush paradigm in the same manner. Facial EMG testing was performed in Linköping, Sweden.

Affective touch testing: psychophysics

Brush strokes were delivered using a goat hair artist's brush. The experimenter was guided regarding brushing velocity by a visual meter on a monitor, not visible to the subject. Six repetitions of each velocity were presented in pseudo-randomized order. In each trial, instructions appeared above the visual-analogue scale to 'rate how pleasant the touch feels to you' followed by a 4–6 s response interval. To ensure that their arm and the experimenter were out of view, participants positioned the stimulated arm behind a curtain or wore goggles flanked by occluders.

Affective touch testing: facial EMG

Using the same settings as for affective touch testing, participants were precluded from receiving any visual cues regarding touch administration. Participants were on one side of a curtain with the left arm extending to the other side of the curtain, where the experimenter administered the brushing. Participants were instructed to look straight ahead at a fixation cross that was presented on a computer screen throughout the brushing trials. In total, each participant completed 32 trials; 16 at each velocity. Participant 3 completed two extra blocks of trials but only self-report ratings were collected during these final blocks due to inadvertent sensor removal. The task was presented using Presentation Software (Neurobehavioral Systems, Berkley, CA, USA). EMG was measured using 4mm Ag/AgCl electrodes and an

8mm gel-filled Ag/AgCl ground sensor on the forehead. Sites were cleaned with alcohol and lightly abraded until impedance was below 20kΩ (measured with a Model 1089 MK III Checktrode; UFI, Morro Bay, CA, USA). EMG signals were amplified 5000x, 10–500Hz band pass filtered, and digitized at 1000 Hz using EMG100C amplifiers and MP150 Data Acquisition System from Biopac Systems (Goleta, CA, USA). Acknowledge software (Biopac Systems) was used to apply a comb band stop filter and signals were then rectified and integrated over 20ms. Trained raters, blinded for experimental conditions, identified and excluded trials with excessive baseline activity or artifactual activations.[8]

Mouse strains

The TH^{CreERT2} mice were purchased from Jackson Labs (Bar harbour, Maine, USA) and have been previously described.[9] The floxed Nav1.7 (Nav1.7flox/flox) and Nav1.8^{Cre} mice have been previously described.[10] The following lines were used as Cre dependent reporter lines, R26-Flox-Stop-TdTomato reporter mice (Ai14) and RCL-ChR2(H134R)/EYFP (Ai32), and were purchased from Jackson labs. C57BL/6 mice were purchased form the Oxford University Breeding Unit.

All experiments were carried out on adult male and female mice. C57BL/6 mice were used for skin-nerve pharmacology and in-situ hybridisation experiments. TH$^{CreERT2+/-}$Ai14$^{+/-}$ mice and Nav1.8$^{Cre+/-}$Ai14$^{+/-}$ mice were used for histological experiments. TH$^{CreERT2+/-}$Ai32$^{+/-}$ mice were used for patch-clamp pharmacology experiments. Intrathecal injections and patch-clamp electrophysiology was conducted on TH$^{CreERT2+/-}$ and TH$^{CreERT2+/-}$Nav1.$^{7flox/flox}$ (TH^{CreERT2}:Nav1.7-KO) mice. All behaviour and skin-nerve electrophysiology was carried out on TH$^{CreERT2-/-}$Nav1.7$^{flox/flox}$ (Nav1.7-WT) and TH$^{CreERT2+/-}$Nav1.7$^{flox/flox}$ (TH^{CreERT2}:Nav1.7-KO) littermate mice.

Immunohistochemistry (IHC)

Briefly, fixed/sectioned samples were washed in PBS and blocked in a blocking solution (5% normal donkey serum, 0.3% TritonX-100, PBS) for 1hr at room temperature (RT). Primary antibodies (Supplementary Table 3) were diluted in blocking solution and applied to tissue or cells overnight at RT. The next day samples were washed in a wash solution (0.3% TritonX-100, PBS) followed by a 2hr incubation with secondary antibodies diluted in wash solution at RT. Samples were mounted using Vectorshield and imaged on a confocal microscope (Zeiss

LSM-710). Images were analysed using Fuji/ImageJ (NIH). For quantification at least three sections per animal were used, with at least 3 animals per group.

In-situ hybridisation (ISH)

ISH was performed using two methods; the first method (related to Fig. 3 and Supplementary Fig. 3C-D) was performed by following the user instructions for the RNAScope2.5 RED Chromogenic assay kit (Advanced Cell Diagnostics). Briefly, tissue was pre-treated using hydrogen peroxide and a protease treatment. Tissue was next incubated for 2hrs at 40°C with a Nav1.7mRNA specific probe (Cat no. 457641). Next, a series of 6 probe amplification steps were carried out followed by a fast red detection step.

The second method of ISH (related to Supplementary Fig. 3B) was performed using digoxigenin labelled probes. Nav1.7 probes were hybridized overnight at 55°C, and the slides incubated with the horseradish peroxidase anti-digoxigenin antibody (Roche). Final detection was achieved using cy3 TSA plus kit (Perkin Elmer). RNA probes were synthesized using gene-specific PCR primers and cDNA templates from adult mouse DRG. The following oligonucleotides were used for *SCN9A* PCR probe synthesis snc9a-F : 5′-GAAGGTGACTCACTCGTG-3′ and snc9a-R : 5′-CATGTGCGCCTGAATTTC-3′.[11] Samples were also co-stained using IHC using a standard IHC protocol. ISH tissue was imaged on a confocal microscope and analysis was carried out on Fuji/imageJ. TH+ cells were selected using Fuji/imageJ and the Nav1.7mRNA fluorescence intensity was measured. Three random regions of the imaged section not containing cells was selected and intensity measured to determine signal background. An intensity cut off (3x average background intensity) was applied to distinguish Nav1.7mRNA positive and negative cells.

Tamoxifen dosing

Nav1.7-WT and TH^{CreERT2}:Nav1.7-KO mice were given tamoxifen dissolved in corn oil (1x 50mg/kg I.P) at 8-10wks of age and behaviour and electrophysiology carried out 4-6 weeks later. This time point was chosen as Shields *et al* (2018) have previously shown that when using Nav1.7$^{flox/flox}$ mice, the Nav1.7 protein can still be detected prior to 3 weeks (but not at 4 weeks) post tamoxifen. TH^{CreERT2}:Ai14 and TH^{CreERT2}:Ai32 and mice were given tamoxifen at 8-10 weeks of age and tissue was taken for histology or electrophysiology at least 2 weeks

later. Animals that received intrathecal injections were tamoxifen dosed 1 week post-surgery to allow simultaneous Nav1.7 KO and eGFP expression.

Mechanical sensory testing

Von Frey testing

Mice were then tested on their plantar hind paws using calibrated von Frey hairs (Linton Instrumentation) using the 'up-down' method (Dixson 1980) to evaluate their 50% paw withdrawal thresholds. Mice were tested on 3 different days to obtain an average baseline value.

Brush/Cotton swab

The plantar hind paws of mice were brushed (1 cm s^{-1}) with a fine artists paint brush or a cotton swab that had been puffed out to 3 times its original size. Each mouse received 5 successive stimuli on alternate hind paws (10s apart), twice. The number of responses were recorded. A response included, lifting, flicking or moving the hind paw or walking away from the stimulus. Mice were tested on 3 different days to obtain an average baseline value.

Sticky tape assay

A small (8mm) sticky tape dot was placed onto the hairy dorsum of the hind paw. Mice were observed for 5 mins. The time taken to notice the tape (sense time) and number of bout attempts to remove the tape, were recorded and analysed.

Tactile acuity (sandpaper) testing

This assay was similar to and adapted from.[12,13] Mice were placed in an black box that with a neutral laminated surface. The black box was (digitally) divided into two halves (side 1 and side 2) and mice were individually video tracked in darkness (box illuminated with only red lamp to aid video tracking). Day 1, mice were tracked for 15 mins to ensure mice equally spent time on both sides. Days 2-3 mice were reintroduced into the chamber with one half of the floor being course (60-Grit) or fine (120-Grit) sandpaper and the other half being a control smooth surface. This was the underside of the sandpaper which was smooth and had the same odour. Mice were individually video tracked for 30 mins and time spent on each surface analysed. Assignment of sandpaper to side 1 or 2 was randomised.

Thermal sensory testing

53ºC Hotplate

Mice were placed onto a Perspex enclosed Hotplate (UgoBasile) and were observed until mice displayed pain behaviours on their hind paws i.e. Lifting, Flicking, licking of the hind paw (cut off 30 s to prevent tissue damage). The latency to respond was recorded and mice were tested on 3 different days to obtain average baseline value.

Thermal Gradient

Mice were able to freely explore a thermal gradient apparatus (BIOSEB) where a metal platform was heated in a gradient from 54ºC to 6ºC. Mice were allowed to freely explore the thermal gradient for 30 mins and their activity was monitored and tracked using a HD webcam and ANYMaze software.

Intrathecal injections

Adult $TH^{CreERT2}$ and $TH^{CreERT2}$:Nav1.7-KO mice received an intrathecal injection (i.t) as previously described.[14] Briefly, mice were anesthetised using 2% isoflurane, a rostral to caudal incision (1-2cm) was made, and the T10-11 vertebra were exposed. Lateral to the midline the dura was identified and carefully punctured with a 30g needle. A polyethelene cannula (designed of connecting tubing of decreasing size until the final cannula tip measured 0.008in (O.D) x 0.004in (I.D)), was prefilled with virus and inserted 1cm caudal into the subdural space. Using a pump system 5µl of AAV9.Flex.eGFP at $1x10^{13}$ vg/ml (VVF, Zurich, Switzerland) was injected at a rate of 1µl/min. The cannula was maintained for 2mins after the viral bolus was delivered to prevent viral backflow. The cannula was removed and the dura sealed using DuraGel (Cambridge, NeuroCare). Finally, the incision site was sutured closed and appropriate post-operative care and analgesics given (local 2 mg/kg Marcain, AstraZeneca and systemic 5 mg/kg Rimadyl, Pfizer).

Dorsal root ganglion (DRG) culture

DRG neuron cultures were prepared as detailed previously.[14] DRG were harvested and enzymatically digested at 37°C for 90min in HBSS without Ca^{2+} and Mg^{2+} (ThermoFisher Scientific) with dispase type II (4.7 mg/ml) and collagenase type II (4 mg/ml) (Worthington Biochemical). Neurons were mechanically dissociated into a single-cell suspension with fire polished glass capillaries and plated in complete Neurobasal® medium [Neurobasal® media supplemented with 2% (v/v) B27 and 1% (v/v) penicillin streptomycin (ThermoFisher Scientific)] onto laminin/poly-D-lysine (BD Biosciences) treated coverslips. Murine growth

factors (50 ng/ml; mouse nerve growth factor, PeproTech and 10 ng/ml; glial-derived neurotrophic factor; Peprotech) were added to the media and cultures used for experiments 24–48 h later.

Single cell “picking”, RT-PCR, and qPCR

All solutions were made in an RNAse-free environment and with 1% DEPC-treated water. Patch pipettes of 2-3 MΩ were pulled from borosilicate glass capillaries (WPI) and filled with ~ 2µl of a standard internal solution: (mM) 100 K-gluconate, 28 KCl, 1 MgCl2, 5 MgATP, 10 HEPES, and 0.5 EGTA. Solution was pH adjusted to 7.3 with KOH and osmolarity adjusted to 305 with glucose. eGFP+ DRG neurons (from at least 3 mice per group) were detected with an Olympus microscope with an inbuilt GFP filter set (470/40x excitation filter, dichroic LP 495 mirror and 525/50 emission filter) and “picked” individually using patch pipettes and negative pressure. Samples were transferred into Eppendorfs containing 2µl resuspension buffer and lysis enhancer (CellDirect One-Step qRT-PCR Kit, ThermoFisher) supplemented with 1 U/µl Ribonuclease Inhibitor (Takara). Negative “no cell” controls - where cell collection was mimicked but only solution was aspirated - were taken throughout sample collection to ensure solutions were not contaminated. Samples were snap frozen immediately prior to downstream processing. After DNAse treatment using manufacturer’s protocol (CellDirect), cDNA was synthesized with SuperScript III First Strand SuperMix (ThermoFisher). Each cell lysate was treated as a double reaction and the manufacturer’s protocol using random hexamers was followed, allowing for multiple PCR reactions from a single cell. cDNA was stored at -20°C until further use.

Prior to Nav1.7 quantification, RT-PCR for βIII tubulin (F primer-GGCCTCCTCTCACAAGTATGT, R primer- CAGGGAATCGAAGGGAGGTG) was performed to confirm each single cell sample was successfully collected. RT-PCRs used a gene-specific preamplification with Platinum Taq DNA Polymerase (ThermoFisher) followed by RedTaq ReadyMix (Sigma). βIII tubulin positive samples and no-cell controls were then used for Nav1.7 qPCR reaction, performed in triplicate (SYBR Green LightCycler 480, Roche). Nav1.7 primers used were those previously published by Shield *et al* 2018, and concentrations were calculated as 2^{-Cp}. Reference genes are not typically used for single cell normalization (due to temporal fluctuations in RNA synthesis), thus Nav1.7 mRNA data were normalized to the mean WT expression for presentation. To verify Nav1.7 specificity, RT-

PCRs were subsequently performed to verify band size, and bands were gel extracted for sequencing (QiaQuick Gel Extraction Kit, Qiagen).

Whole-cell patch clamp solutions

Filamental borosilicate glass capillaries (1.5 mm OD, 0.84 mm ID; World Precision Instruments) were pulled to form patch pipettes of 2–4 MΩ tip resistance and filled with an internal solution containing (mM): 140 CsF, 10 NaCl, 1 EGTA and 10 HEPES; pH was adjusted to 7.3 with CsOH and osmolarity set to 300 mOsm. The extracellular solution contained (mM): 70 NaCl, 50 N-methyl-d-glucamine, 20 Tetraethylammonium chloride, 1 CaCl2, 3 KCl, 1 MgCl2, 10 HEPES, 10 Glucose and 0.1 CdCl2; pH was adjusted to 7.3 with NaOH and osmolarity set to 310 mOsm.

Ex-vivo skin nerve preparation - stimuli

C-LTMR receptive fields were stimulated using a piezo electric stimulator (Physik Instrument) in conjunction with a force transducer (Kleindiek). To measure stimulus response functions, the receptive fields were stimulated using an increasing stimulus force protocol and/or an increasing stimulus velocity protocol. Thermal testing was achieved using a custom built receptive field isolator (Dr. Roberto De Col) that allowed pump-driven, temperature controlled, buffer to be delivered to and restricted to the C-LTMR receptive field. Three successive thermal ramps were applied 31-14°C, 14-42°C, 42-14°C, using a single channel bipolar temperature controller (Warner instruments CL-100) with an in line heater/cooler (Warner instruments SC-20). Note: we believe the differences between the WT stimulus responses values in Figure 5J and Figure 6F are due to different experimental designs. The recordings in Figure 6 are much longer, and require repeated testing while the receptive field is isolated. We believe a technical limitation (in our hands) is that C-LTMRs are very sensitive afferents that exhibit rundown/ partial exhaustion during long, isolated, repeated recordings. Something, which is particularly apparent during supra-threshold testing. These observations are consistent with extensive animal evidence suggesting that C-LTMRs experience fatigue.[15–17] In addition, from microneurography in C-Tactile afferents in humans, CLTMR fatigue has been demonstrated.[18–20] However, (and importantly) in figure 6, recording conditions were consistent between

vehicle and PF-05089771, therefore we believe any observed run down is not responsible for the effect of the blocker which showed a marked difference to vehicle.

Computational modelling of C-LTMRs

Briefly, the C-LTMR model was executed using the NEURON simulation environment. The model was constructed as such the membrane capacitance (Cm) was 12pF and contained the voltage-gated ion channels; Nav1.7, Nav1.8, Kv1, Kv2, Kv3, Kv4 and a leak channel. The voltage dependence and current kinetics of each ion channel in the model was described by Zheng *et al* (2019). The maximal conductance ($\bar{g}$) for each ion channel is as follows: Nav1.7 (30 mS/cm2), Nav1.8 (40 mS/cm2), Kv1 (0.06 mS/cm2), Kv2 (2 mS/cm2), Kv3 (0.05 mS/cm2) and Kv4 (11 mS/cm2). The model also included a non-voltage dependent leak conductance (0.02 mS/cm2) with a reversal potential of -80mV.

1. Nagi SS, Marshall AG, Makdani A, et al. An ultrafast system for signaling mechanical pain in human skin. *Sci Adv*. 2019;5(7):eaaw1297. doi:10.1126/sciadv.aaw1297
2. Rolke R, Baron R, Maier C, et al. Quantitative sensory testing in the German Research Network on Neuropathic Pain (DFNS): Standardized protocol and reference values. *Pain*. 2006;123(3):231-243. doi:10.1016/j.pain.2006.01.041
3. McDermott LA, Weir GA, Themistocleous AC, et al. Defining the Functional Role of NaV1.7 in Human Nociception. *Neuron*. 2019;101(5):905-919.e8. doi:10.1016/J.NEURON.2019.01.047
4. Staud R, Price DD, Janicke D, et al. Two novel mutations of SCN9A (Nav1.7) are associated with partial congenital insensitivity to pain. *Eur J Pain*. 2011;15(3):223-230. doi:10.1016/j.ejpain.2010.07.003
5. Bogdanova-Mihaylova P, Alexander MD, Murphy RPJ, Murphy SM. SCN9A-associated congenital insensitivity to pain and anosmia in an Irish patient. *J Peripher Nerv Syst*. 2015;20(2):86-87. doi:10.1111/jns.12123
6. Ramirez JD, Habib AM, Cox JJ, et al. Null mutation in SCN9A in which noxious stimuli can be detected in the absence of pain. *Neurology*. 2014;83(17):1577-1580. doi:10.1212/WNL.0000000000000913
7. Weiss J, Pyrski M, Jacobi E, et al. Loss-of-function mutations in sodium channel Nav1.7 cause anosmia. *Nature*. 2011;472(7342):186-190. doi:10.1038/nature09975
8. Fridlund AJ, Cacioppo JT. Guidelines for Human Electromyographic Research.

Psychophysiology. 1986;23(5):567-589. doi:10.1111/j.1469-8986.1986.tb00676.x

9. Abraira VE, Kuehn ED, Chirila AM, et al. The Cellular and Synaptic Architecture of the Mechanosensory Dorsal Horn. *Cell*. 2017;168(1-2):295-310.e19. doi:10.1016/J.CELL.2016.12.010
10. Nassar MA, Stirling LC, Forlani G, et al. Nociceptor-specific gene deletion reveals a major role for Nav1.7 (PN1) in acute and inflammatory pain. *Proc Natl Acad Sci*. 2004;101(34):12706-12711. doi:10.1073/pnas.0404915101
11. Ahmad S, Dahllund L, Eriksson AB, et al. A stop codon mutation in SCN9A causes lack of pain sensation. *Hum Mol Genet*. 2007;16(17):2114-2121. doi:10.1093/hmg/ddm160
12. Wetzel C, Hu J, Riethmacher D, et al. A stomatin-domain protein essential for touch sensation in the mouse. *Nature*. 2007;445(7124):206-209. doi:10.1038/nature05394
13. Maricich SM, Morrison KM, Mathes EL, Brewer BM. Rodents rely on Merkel cells for texture discrimination tasks. *J Neurosci*. 2012;32(10):3296-3300. doi:10.1523/JNEUROSCI.5307-11.2012
14. Weir GA, Middleton SJ, Clark AJ, et al. Using an engineered glutamate-gated chloride channel to silence sensory neurons and treat neuropathic pain at the source. *Brain*. 2017;140(10):2570-2585. doi:10.1093/brain/awx201
15. Iggo A. Cutaneous mechanoreceptors with afferent C fibres. *J Physiol*. 1960;152(2):337-353. doi:10.1113/jphysiol.1960.sp006491
16. Bessou P, Burgess PR, Perl ER, Taylor CB. Dynamic properties of mechanoreceptors with unmyelinated (C) fibers. *J Neurophysiol*. 1971;34(1):116-131. doi:10.1152/jn.1971.34.1.116
17. Iggo A, Kornhuber HH. A quantitative study of C-mechanoreceptors in hairy skin of the cat. *J Physiol*. 1977;271(2):549-565. doi:10.1113/jphysiol.1977.sp012014
18. Nordin M. Low-threshold mechanoreceptive and nociceptive units with unmyelinated (C) fibres in the human supraorbital nerve. *J Physiol*. 1990;426(1):229-240. doi:10.1113/jphysiol.1990.sp018135
19. Vallbo ÅB, Olausson H, Wessberg J. Unmyelinated Afferents Constitute a Second System Coding Tactile Stimuli of the Human Hairy Skin. *J Neurophysiol*. 1999;81(6):2753-2763. doi:10.1152/jn.1999.81.6.2753
20. Pitcher M, Le Pichon CE, Chesler A. Functional properties of c-low threshold mechanoreceptors (C-LTMRs) in nonhuman mammals. *Affect Touch Neurophysiol CT Afferents*. 2016:31-48. doi:10.1007/978-1-4939-6418-5_2
